# Transcriptomic Signature of *Leishmania* Infected Mice Macrophages: A Metabolic Point of View

**DOI:** 10.1371/journal.pntd.0001763

**Published:** 2012-08-21

**Authors:** Imen Rabhi, Sameh Rabhi, Rym Ben-Othman, Axel Rasche, Sysco Consortium, Adriani Daskalaki, Bernadette Trentin, David Piquemal, Béatrice Regnault, Albert Descoteaux, Lamia Guizani-Tabbane

**Affiliations:** 1 Laboratory of Immunopathology, Vaccinology and Molecular Genetics (LIVGM), WHO Collaborating Center for Research and Training in Leishmaniasis and Laboratoire International Associé Ingenierie Biomoléculaire (LIA-CNRS), Institut Pasteur de Tunis, Tunis-Belvedere, Tunisia; 2 Department Lehrach (Vertebrate Genomics Department), Max-Planck-Institute for Molecular Genetics, Berlin, Germany; 3 Skuldtech. Cap Delta - ZAC Euromedecine II, Grabels, France; 4 Genopole, Institut Pasteur de Paris, Paris, France; 5 INRS-Institut Armand Frappier and Centre for Host-Parasite Interactions, Laval, Québec, Canada; Instituto Oswaldo Cruz, Fiocruz, Brazil

## Abstract

We analyzed the transcriptional signatures of mouse bone marrow-derived macrophages at different times after infection with promastigotes of the protozoan parasite *Leishmania major*. Ingenuity Pathway Analysis revealed that the macrophage metabolic pathways including carbohydrate and lipid metabolisms were among the most altered pathways at later time points of infection. Indeed, *L. major* promastiogtes induced increased mRNA levels of the glucose transporter and almost all of the genes associated with glycolysis and lactate dehydrogenase, suggesting a shift to anaerobic glycolysis. On the other hand, *L. major* promastigotes enhanced the expression of scavenger receptors involved in the uptake of Low-Density Lipoprotein (LDL), inhibited the expression of genes coding for proteins regulating cholesterol efflux, and induced the synthesis of triacylglycerides. These data suggested that *Leishmania* infection disturbs cholesterol and triglycerides homeostasis and may lead to cholesterol accumulation and foam cell formation. Using Filipin and Bodipy staining, we showed cholesterol and triglycerides accumulation in infected macrophages. Moreover, Bodipy-positive lipid droplets accumulated in close proximity to parasitophorous vacuoles, suggesting that intracellular *L. major* may take advantage of these organelles as high-energy substrate sources. While the effect of infection on cholesterol accumulation and lipid droplet formation was independent on parasite development, our data indicate that anaerobic glycolysis is actively induced by *L. major* during the establishment of infection.

## Author Summary

*Leishmania* are obligated intracellular pathogens that develop almost exclusively in macrophages. Experimental leishmaniasis in mice is one of the most extensively studied models of intracellular infections both at the level of the parasite and host immune responses. We took advantage of Balb/c mice model to investigate gene expression profile through Affymetrix oligonucleotide arrays. In order to have a general and dynamic picture of the complex biological events that are acting in the context of *Leishmania* intracellular parasitism, we investigated the mouse macrophage response to initial invasion of *L. major* over a time course that extended from one to 24 hours post-infection. Our results reveal the alteration of several biological processes and metabolic changes. Indeed, similarly to different other pathogens, *Leishmania* induces cholesterol accumulation and foam cell formation that have been confirmed by confocal microscopy experiments. Whether *Leishmania* parasites take advantage of this high-energy source is now under investigation. Our findings provided further understandings in host responses to *Leishmania* infection.

## Introduction

*Leishmania*, the causative agent of vector-borne diseases, known as leishmaniases, lives as an obligate intracellular parasite within mammalian hosts. The outcome of infection depends largely on the activation status of macrophages, the first line of mammalian defense and the major target cells for parasite replication. Understanding the strategies developed by the parasite to circumvent the macrophage defense mechanisms and to survive within these cells may help defining novel therapeutic approaches for leishmaniases. High-throughput techniques have allowed the simultaneous identification and analysis of thousands of genes or proteins modulated in response to host-pathogen interaction. Different previous studies have used microarray technology to investigate the responses of macrophages from human and mouse origins to *Leishmania* infection [1], [2], [3], [4]. Most of these studies have dealt essentially with established infection, and limited responses to various species of *Leishmania* were observed. To obtain a dynamic and informative picture of macrophage behaviour in response to *Leishmania* promastigotes, we investigated the mouse macrophage response to initial invasion of *L. major* over a time course that extended from one to 24 hours post-infection. As controls, we used heat-killed promastigotes infected macrophages to determine the genes and pathways actively regulated by *Leishmania* parasites. Transcripts significantly modulated by *Leishmania* infection over time were identified and a subset of these genes confirmed by reverse- transcription quantitative real-time PCR (RT-qPCR). Hierarchical Clustering was performed to identify gross gene expression features and Ingenuity Pathway Analysis (IPA) was used to flag the mouse biological pathways, networks, and functions significantly altered by *Leishmania* infection during the first 24 hours post-infection.

Analysis of the microarray data presented here revealed that in addition to oxidative stress, immune responses, and inflammatory genes that have been widely described in previous works, the lipids and carbohydrates metabolic pathways are among the most relevant biological networks fitting our data set, modulated by *L. major* infection. Among those, anaerobic glycolysis was identified as one of the major pathway actively regulated by the parasite.

## Materials and Methods

### Parasites

Promastigotes of the *L. major* tunisian strain GLC94 (MHOM/TN/95/GLC94 zymodeme MON25) were grown at 26°C in RPMI 1640, supplemented with 5 mM L-glutamine, 10% heat inactivated foetal calf serum (Perbio science, Brebières, France), penicillin (100 U/ml) and streptomycin (100 µg/ml). Metacyclic rich fraction obtained using Ficoll gradient was used in all experiments. Briefly, stationary phase cultures of *Leishmania* were centrifuged at 5 000 g for 10 min at room temperature and resuspended in 2 ml of PBS. The cell suspensions were then loaded onto a Ficoll gradient composed, from the bottom of 2 ml of 20%, 5 ml of 10% and 5 ml of 5% Ficoll diluted in PBS. The gradient was next centrifuged at 1 300 *g* for 10 min at room temperature. The metacyclic promastigotes were recovered on the top of 10% Ficoll layer.

### Cells isolation and culture

BALB/c mice (Elevage Janvier) were killed and hind legs removed for bone marrow derived macrophages (BMDM) isolation. Briefly, femurs and tibias were flushed with RPMI 1640 using a 25-gauge needle. Contaminating erythrocytes were lysed through the addition of Gey lysis solution (ammonium chloride 1.5M, EDTA 0.1 mM, pH 7.3). All cells were incubated in T75 culture flasks at 1.5 10^6^ cell per ml in RPMI 1640 media supplemented with 5 mM L-glutamine, 10% heat inactivated foetal calf serum, penicillin (100 U/ml) and streptomycin (100 µg/ml) and 80 ng/ml M-CSF (Peprotech, Neuilly sur Seine, France) overnight for stromal cell elimination. Non-adherent, immature macrophages were transferred to fresh culture-treated Petri dishes (Nunc, USA) and grown for 7 days, with re-feeding on day 3, to induce macrophage differentiation. Generated macrophages were assessed by flow cytometry for expression of F4/80 (around 90% were positive).

### Ethics statement

All mouse work was done according to the directive 86/609/EEC of the European parliament and of the council on the protection of animals used for scientific purposes. Approval for *mice* experiments was obtained from the ethic committee of Institute Pasteur of Tunis with ethics approval number 1204.

### Cells infection

BMdM were incubated at a parasite to cell ratio of approximately 10∶1 with Ficoll purified metacyclic promastigotes of *L. major*. After the desired time of incubation at 37°C in 5% CO2, non-ingested parasites were removed and the cells were harvested to prepare samples. Standard Giemsa staining was used to determine the percentage of infected cells and to insure for homogenate cell infection under the different conditions.

### RNA isolation, microarray hybridization and analysis

Macrophages were lysed directly in 1 ml Trizol reagent (Invitrogen). Total RNA from uninfected and infected macrophages were prepared using the RNeasy mini kit (Qiagen) and treated with DNase according to the manufacturer's protocol. Extracted RNAs were stored at −80°C. RNAs were quantified using NanoDrop ND-1000 micro-spectrophotometer and RNA quality was monitored on Agilent RNA Pico LabChips (Agilent Technologies, Palo Alto, CA). 100 ng of RNA from each biological condition were amplified and labelled with biotin according to the GeneChip whole transcript sense target labeling assay manual and using the GeneChip WT cDNA Synthesis and amplification Kit and WT terminal labeling Kit. The fragmented ssDNA was checked on Agilent RNA Pico LabChips. The fragmented and labeled ssDNA was hybridized to the GeneChip Mouse Gene 1.0 ST array (Affymetrix, Santa Clara, CA), washed with the Fluidics station 450 and scanned using the Affymetrix Scanner 30007G. QC analysis was performed before and after normalization using BoxPlot of total intensities, MAPlots for all replicates and PCAplots. All microarrays of this study passed the quality control.

Cell intensity files were generated with GeneChipOperating Software (GCOS). Each infection and control time points were performed on three different samples, using different preparations of BMdMs, and processed independently to give three biological replicates.

### Normalization

The data preprocessing step included intrachip and interchip normalisation and summarisation. The intrachip normalisation step corrects for the GC content of the probes, the interchip normalisation step reduces non-biological differences between chips and the summarisation step combines the probe intensities into single gene expression values.

The data from BALB/c mouse are different to classical 3′-type Affymetrix chips as mismatch probe sets are not available. Since the annotation of the new “exon-like” mouse Affymetrix chip (Affymetrix/MoGene-1_0-st-v1) was not present in all databases, IDs had to be re-mapped on their Ensembl gene. The process is implemented completely in R (Version R-2.7.0) with the use of several BioConductor (BioC 2.2) packages [5].

Probe sets are defined each by one Entrez gene. If the probe sequence matches uniquely to the gene and no SNP hits the probe segment of the DNA the probe is assigned to the Entrez gene. We used a probe-Entrez gene assignment from version 11.0.0 of Dai et al. [6]. (Entrez database was downloaded for human from 11.03.2008 and for mouse from 28.06.2008). After summarisation the Entrez identifer were directly mapped to Ensembl gene. For this mapping BioMart/EnsMart was used via the biomaRt package [7]–[9] in Ensembl v50. The processing relies on Ensembl genes and it is straightforward to use a probe-Ensembl gene assignment. At the time of processing, no assignment for HuGene and MoGene arrays to Ensembl genes was available from [6].

For the intrachip normalisation the ‘Model-based Analysis of Tiling-arrays’ (MAT) was implemented, similar to [10], since this method provides the most advanced GC correction for whole-transcript prepared samples. MAT is a probe affinity model which combines content and position dependency of probe sequences in a unified linear model. The parameters of the model are estimated from the control probes and subsequently the probe affinities are calculated for the perfect matches. On linear intensity scale, the probe intensities are divided by an estimated probe affinity. As a second step, we applied an interchip normalisation in form of a quantile normalisation to adjust the intensity distributions over the arrays, as this kind of normalisation appeared successful to reduce unwanted effects between 3′ expression arrays [11]. As a last step in the preprocessing of the data we applied a summarisation of probe intensities to a probe set expression. In this process, a median is computed over the intensities in two replicate dimensions: a) The different probe intensities within the probe set; b) The arrays with the same biological condition. This provides very robust summarised expression values also for low-replicate settings.

Detection call p-values were computed for each probeset with a paired Wilcoxon signed rank test that compares probe intensity to control probes of similar GC content. More precisely, each probe is compared to the 75% quantile of the set of control probes with similar GC content. A gene probeset was called present when the corresponding FDR corrected p-value was below 5%. The sole threshold in this approach is the height of the quantile (75%) in the GC bin. The same probe-Entrez gene assignment and subsequent mapping was used as in the summarisation process.

Expression analysis used the R Bioconductor package Limma to identify genes that met statistical (*P*<0.05 after adjustment according to the method of Benjamini and Hochberg and fold-change criteria (at least a 1.5-fold change) for differential expression using the following contrasts: macrophages infected with live parasites at a given time point versus non infected macrophages incubated with vehicule (media) for the same time. The same contrast was used for heat-killed *Leishmania*-infected macrophages. Macrophage genes modulated during the kinetics were first detected.

In accordance with MIAME (Minimum Information About a Microarray Experiments) regulations, all data were deposited into GEO (Gene Expression Omnibus) database at www.ncbi.nlm.nih.gov/geo/ under the accession number GSE31995.

### Ingenuity global functional analysis

Ingenuity Pathways Analysis (IPA; Ingenuity, CA; Systems 2008) is derived from known functions and interactions of genes published in the literature as well a set of canonical pathways and cellular Toxic molecules markers in the context of several studied diseases.

The Ingenuity IPA Tool was used to identify the most significant macrophagic biological networks, cellular functions and canonical pathways altered by *Leishmania* parasite infection, based on a Fischer's exact test to calculate a p-value for each biological function founded (at least *P*<0.01).

A list of the statistically significant differential genes expression in *L. major*-infected BALB/c macrophage for each time points of the kinetic was generated and mapped to their functional networks in the IPA database and ranked by score. The relationships between the generated networks and known pathways were then investigated using the canonical pathway analysis function. In addition, we applied 2 IPA analysis methods: an analysis of dataset corresponding to each time point of infection and a global analysis along the kinetic of infection that allowed us to identify the Top canonical and cellular pathways altered across all time points of the infection.

### dchip clustering

The Affymetrix 1.07 array data were analyzed using dChip software (http://www.biostat.harvard.edu/~cli/dchip_2010_01.exe) in order to identify the marker genes cluters regulated later or earlier throughout the kinetic of *Leishmania* infection. Moreover, the dchip software was used to check the signature of live *Leismania major* parasite infection comparing with the Killed parasite one.

### Quantitative real time PCR

RNA quantity, was controlled using NanoDrop ND-1000 micro-spectrophotometer and RNA quality and integrity (RNA Integrity Number, RIN>9) was monitored on Agilent RNA Pico LabChips (Agilent Technologies, Palo Alto, CA). Reverse transcriptions were performed for each of 96 mice samples in 20 µl final reaction volume with 273 ng of total RNA using 200 Units of SuperScript III enzyme (M-MLV RT, Invitrogen) and 250 ng of random primers according to manufacturer's instructions (25°C 10 min, 42°C 50 min, 70°C 15 min). All RT reactions were performed the same day with same pipetor set and same manipulator. A negative control was included by performing a RT with no template. qPCR experiments were carried out using EVA Green chemistry on BioMark qPCR apparatus (Fluidigm) following manufacturer's instructions. For each cDNA sample, a Specific Target Amplifications (STA) was performed with a pool of primers targeting all selected genes (Pre-Amplification of 14 cycles using TaqMan PreAmp Master Mix (Applied Biosystems) and following manufacturer's instructions): Each qPCR was performed with 1/20 STA dilution, in duplicate. Relative gene expression kinetics was created by a first normalization with 4 reference genes followed by a second normalization with Non Infected macrophage cells (NI). Values are expressed in fold changes (2−Delta Delta Ct Method) [12] compared to NI macrophage cells.

### Western blot analysis

Cells extracts were obtained by adding 25 µl of lysis buffer containing 10 mM Tris-HCL pH 7,5, 50 mM NaCl, 50 mM sodium fluoride (NaF), 2 mM EDTA, 1 mM ethylene glycol bis(β-aminoethyl ether)-N.N;N′.N′-tetraacetic acid (EGTA), 2% Nonidet-P40 (NP-40), 0,75% sodium deoxycholate (DOC), 1 mM orthovanadate, 1 µg/ml aprotinine, 1 mM PMSF, 1 mM DTT. After 30 min incubation on ice, the extracts were centrifuged at 15000 rpm for 20 min. 25 µg of whole cell lysates were resolved by electrophoresis in a 12% SDS-polyacrylamide gel. Resolved proteins were electrophoretically transferred onto PVDF sheets (Hybond-P; Amersham) and membranes were blocked by incubation in 3% non-fat milk and 0,1%Tween in PBS for 1 h at room temperature followed by incubation with COX-2 antibody (BD Biosciences, France) in 0,1% tween-PBS. Bound antibody was detected by incubation with horseradish peroxidase-coupled secondary antibody (Amersham Pharmacia Biotech., Buckinghamshire, U.K.). To ensure for equal loading, the blots were then stripped (62.5 mM Tris (pH 6,8), 0,1M β-mercaptoethanol, 2%SDS) and re-probed with ERK1/2, antibody.

### Confocal immunofluorescence microscopy

All incubations and washes were done in 1× PBS. Macrophages were fixed in 4% paraformaldhehyde for 10 min at room temperature.

For Lipid Droplet Staining, Bodipy 493/503 (Molecular Probes) was used as previously described with some modifications [13]. Briefly, cells were stained for 30 min at room temperature using a solution of Bodipy and DRAQ5 (Biostatus, Leicestershire, UK) that allows the staining of macrophage and parasite nuclei. All coverslips were mounted on slides with Fluoromount-G (Southern Biotechnology Associates).

Otherwise, for free cholesterol accumulation, Filipin staining was used as previously described with some modifications [14], [15]. Coverslips were incubated in Glycine to quench paraformaldehyde and cells were then stained with Filipin and DRAQ5 for 2 h at room temperature. All coverslips were washed and mounted on slides with Fluoromount- G.

Detailed analysis of lipid droplet accumulation was performed using an oil immersion Nikon Plan Apo 100 (N.A. 1.4) objective mounted on a Nikon Eclipse E800 microscope equipped with a Bio-Rad Radiance 2000 confocal imaging system (Bio-Rad, Zeiss). Analysis of Filipin staining was visualized with the Eclipse TE 2000-U epifluorescence microscope equipped with Lambda DG-4 illumination. Intensity differences in Filipin staining were quantified using linescan analysis (Metamorph software).

## Results

To investigate the temporal response of macrophages following infection with *L. major* promastigotes, a time course was performed during which RNA was extracted from BMdM infected for 1, 3, 6, 12, and 24 h with live or heat-inactivated promastigotes. GeneChip Mouse Gene 1.0 ST arrays were used to analyse global changes in gene transcripts to generate a pool of genes that was statistically significant (p-value<0.05) and a fold change cut-off of 1.5 in at least one of the five infected samples versus non-infected samples. Of the 18 899 genes represented on the array, our analysis of mRNA expression in mouse BMdM infected by live parasites showed that a total of 782 genes were expressed differentially over the time course while only 375 genes were differentially modulated by macrophages infected by heat-inactivated promastigotes. The relative expression of 83 selected differentially expressed genes from the microarray data was further examined by RT-qPCR on the same samples that those analyzed by microarray analysis. The data from RT-qPCR analysis (Table S3) is consistent with the results obtained by microarrays, albeit with magnitudes different from, and often higher than, those recorded by the microarray analysis.

**Figure 1:**
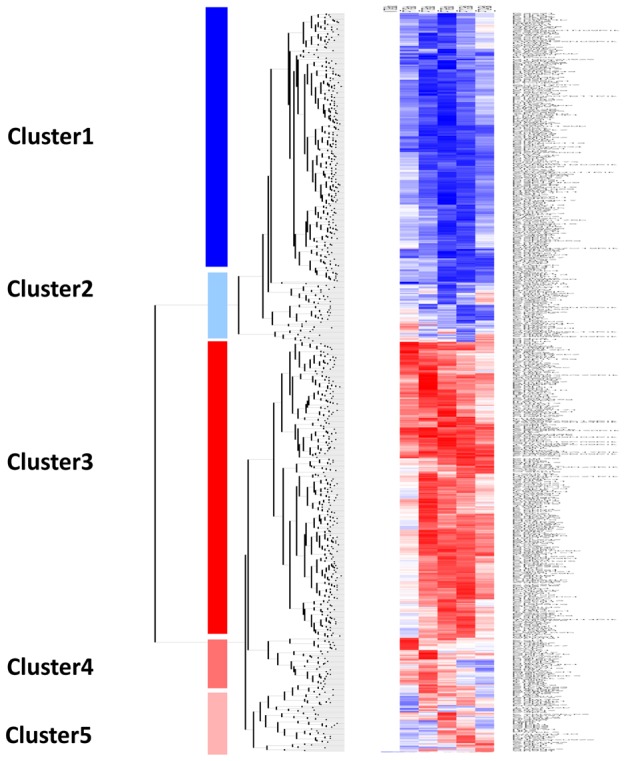
Hierarchical clustering of genes differentially expressed in *Leishmania major* infected BALB/c macrophages. Five distinct clusters were identified by hierarchical clustering analysis using dChip software. Two clusters represent genes that were significantly down-regulated (cluster 1) and up-regulated (cluster 3) over the time course. The second (cluster 2), contains genes that start to be up-regulated from 6 to 24 hours after infection Two other clusters contain genes up-regulated during the early hours (1,3 and 6 hrs) of infection (cluster 4) and at 6, 12 and 24 hrs (cluster 5). Each row represents a spot on the microarray and each column a separate microarray. BMdM response was studied at five different time points (from the left to the right: P-T1h, P-T3h, P-T6h, P-T12h and P-T24h). The left-hand column show non-infected cells (NI) that were used as internal control.

**Figure 2:**
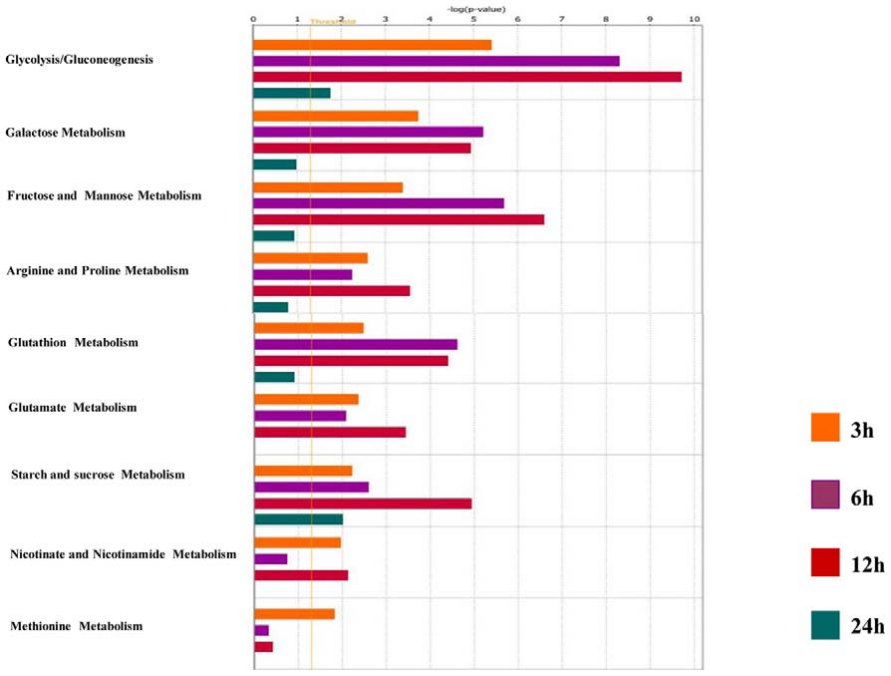
Canonical Metabolic pathways identified by Inguenuity Pathway Analysis software as significantly altered (p<0.05) in *L. major* infected BALB/c macrophages for each time point (3 h (orange), 6 h (violet), 12 h (red) and 24 hours post-infection (green)). The negative log_10_(p-value) are plotted on the Y-axis.

Clustering between the control (non-infected) and the group of the 782 genes (modulated by live parasites) was carried out and shows that these genes can be grouped into five different clusters (Figure 1). Cluster 1, contained a set of down-regulated genes while cluster 3 included a set of genes up-regulated across time course of experiment. Cluster 4 contained genes up-regulated during the first hours of infection while cluster 5 included genes up-regulated late during the infection. Several of those genes have been pinpointed by others and reported to be modulated by different *Leishmania* species [1], [2], [4]. These included different genes coding for chemokines and chemokines receptors such as CCL4 and CCR2, cytokines such as IL-1β, transcription factors such as c-*fos* and NF-κB, and receptors such as TLR2. Further analysis using Ingenuity Pathways Analysis (Ingenuity Systems, Redwood City, CA; http://www.ingenuity.com) (IPA) of these differentially expressed genes revealed that besides the induction of transcriptional responses to oxidative stress, immune responses, and inflammation, canonical metabolic pathways are among the most relevant biological networks fitting our data sets that were modulated by *L. major* infection (Figure 2 and Table S1).

### Immune response and inflammation

Our data show that the transcription of different cytokines and chemokines involved in the inflammatory response and cellular recruitment to the site of inflammation was significantly altered. Indeed, transcription of CXCL1 (up to 3 fold increase), CXCL2 (up to 3.9 fold) and CXCL3 (up to 2 fold) (alias respectively GROα, GROβ, GROγ) was rapidly induced and persisted during the first 12 hours of infection. Consistent with previous studies [2], [16] and associated with the classical activation M1 phenotype, transcription of TNFα (up to 5), CXCL10 (IP10), CCL2 (MIP1α), and other chemotactic cytokines such as CCL3 (up to 6.8) and CCL4 was upregulated following infection. By contrast, transcription of CCR2, the CCL2 receptor, was down-regulated while the mRNA expression of CCRL2 was strongly up-regulated. *Leishmania* infection also led to mRNA downregulation of other cytokines such as IL-1β. Moreover, M2 polarization-associated anti-inflammatory cytokines including IL-1Ra, and receptors such as CD36 were up-regulated in infected cells. Finally, transcription of both NOS2 (which characterizes M1 macrophage phenotype) (up to 5 fold) and arginase1 (ARG1) (which characterize M2 macrophage phenotype) (up to 4 fold) was increased in response to *L. major* promastigotes at different time points. Importantly, both NOS2 and ARG1 were actively induced by live parasite. Indeed, as assessed by qPCR, both genes were not transcribed in heat-killed promastigote-infected cells (data not shown). Infection also induced the expression of co-stimulatory molecules such CD40, CD83, CD86, adhesion molecules (CD38, Itga5, and ICAM1), and tissue invasion molecules such as MMP12 and MMP14.

### Energy, carbohydrate, and lipid metabolism

Among the most relevant pathways modulated by *L. major* infection, we found different metabolic pathways (Figure 2) including glycolysis, gluconeogenesis, tricarboxylic cycle, oxidative phosphorylation and pentose phosphate shunt.

#### *Leishmania* infection induces activation of glycolysis and inhibition of Tricarboxylic cycle (TCA) and oxidative phosphorylation

**Figure 3:**
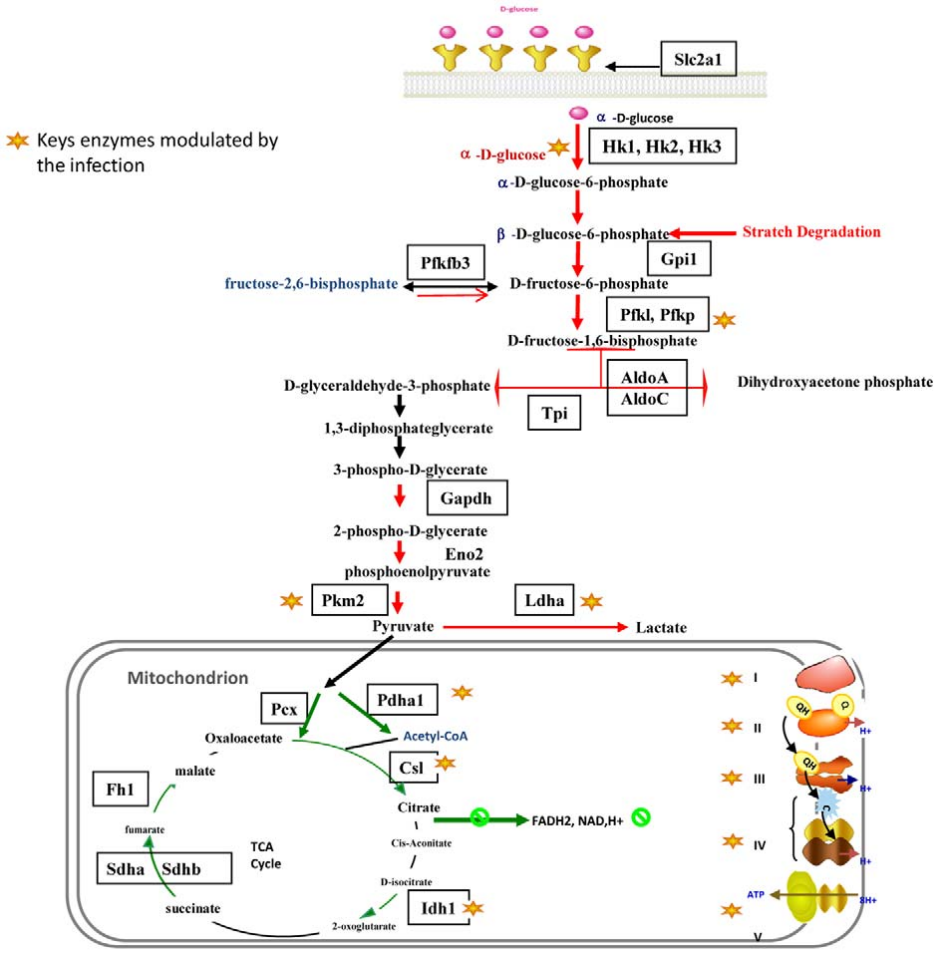
*L. major*-infected macrophages display a modulation of several genes involved in glycolysis, TCA cycle and mitochondrial respiration.

Starting from 3 hours post-infection, almost all the genes encoding host glycolytic enzymes (Figure 3) displayed significant increased expression. This up-regulation concerned the glucose phosphate isomerase (Gpi) (up to 2.5 fold), the fructose-biphosphate aldolase (Aldoa,c) (2 fold), the triose phosphate isomerase 1 (Tpi) (3.6 fold), the glyceraldehyde- 3-phosphate dehydrogenase (2.9 fold), and the enolase 2 (Eno2) (more than 3 fold). Moreover, transcription of the genes encoding the three enzymes that control the rate of glycolysis (the hexokinases (Hk1, 2, 3), the phosphofructokinase (PFK) (2.3 fold), and the pyruvate kinase (Pkm2) (2 fold) was significantly enhanced. On the other hand, transcription of the gene encoding the 6-phosphofructo-2-kinase/fructose-2,6-biphosphatase-3 (PFKFB3), responsible for fructose-2,6 biphosphate generation (the powerful allosteric regulator of PFK) and thus a glycolysis promoting enzyme, was significantly increased.

*Leishmania* infection also induced transcription of genes implicated in the starch degradation pathway. Indeed, the genes encoding the 1,4-alpha-glucan-branching enzyme (Gbe1), the hexokinases (Hk1, Hk2 and Hk3), and the phosphoglucomutases (pgm2 and pgm5) implicated respectively in the fructose-6 and glucose-6 isomerisation, were up-regulated throughout the time course, and this may lead to the feeding of glycolysis. Finally, infection resulted in a robust and sustained increase of the glucose transporter (Slc2a1) mRNA levels (more than 9 fold). Taken together these data suggest that infection with *L. major* promastigotes induces an enhanced uptake of glucose and an increased rate of glycolysis.

Pyruvate generated by glycolysis may be further metabolized to acetyl-CoA by the pyruvate dehydrogenase complex or to lactate by the activation of lactate dehydrogenase (Figure 3). While the microarray data showed that expression of the pyruvate dehydrogenase (Pdh) complex genes was not significantly changed, the data obtained by qPCR showed a downregulation of Pdha1 expression during the first 12 hours post infection (hpi). By contrast, starting from 3 hpi, *Leishmania* induced the up-regulation of the gene encoding the pyruvate dehydrogenase kinase isoenzyme 1 (Pdk1), known to inactivate pyruvate dehydrogenase. In parallel, a sustained activation of the *lactate dehydrogenase-A* transcription was detected by microarray and confirmed by qPCR data. Taken together, these results suggest that *Leishmania* stimulates the catabolism of carbohydrates and induces the host cells to shift to anaerobic glycolysis.

Besides the up-regulation of genes involved in anaerobic glycolysis, the microarray data showed that among the genes encoding components of the TCA (tricarboxylic acid) cycle, transcription of three enzymes-encoding genes, the isocitrate dehydrogenase (Idh1), the succinate dehydrogenase (Sdhb) and the fumarate hydratase (Fh1), was downregulated during infection (Figure 3). Real time qPCR confirmed these findings and showed that besides these three genes, the transcription of two other key enzymes, the citrate synthase like (Csl) and the pyruvate carboxylase (Pcx), was down-regulated throughout the infection time course (Table S3).

The expression of twenty five genes involved in mitochondrial respiration was detected by Affymetrix hybridization (Table S2 and S3) and showed a coordinated down-regulation starting from 12 hpi (Table S2). Indeed, the expression of nine genes coding for Complex I (NADH-UQ-dehydrogenase) subunits decreased by up to 2 fold starting from 12 hpi. Moreover, the qPCR data showed that the mRNA expression of Ndufs4 protein subunits involved in the regulation of Complex I activity was down-regulated at all time points (Table S3). RT-qPCR data also showed that transcription of Sdha and Sdhb genes, coding for respectively the α and β subunits of the Complex II (Succinate UQ dehydrogenase), was down-regulated over the infection time course (Table S3). Moreover, the transcriptional expression of three genes, Uqcrfs1, Uqcrh and Uqcr10, which are involved in the Complex III (cytochrome b/c1 oxidoreductase) activity and structure, decreased slightly starting from 6 hpi. Though modest, this decrease was consistent across several time points. On the other hand, the expression of five genes (Cox17, Cox5a, Cox6a1, Cox6b1, Cox4i1, Cox7a2l) part of the Complex IV (Cytochrome C oxidase), was also down-regulated starting from 12 hpi (Tables S2 and S3). Finally, transcription of two subunits (Atp5e, Atp5g3) of the complex V (ATP-synthase), which couples the proton gradient generated by the respiratory chain to ATP synthesis, decreased starting from 12 hpi (Tables S2 and S3). Taken together, these results show a coordinated transcriptional decrease of an important number of the genes involved in the TCA cycle and the oxidative phosphorylation.

#### *Leishmania* infection modulates the lipid pathway by inducing cholesterol accumulation and triglycerides synthesis

**Figure 4:**
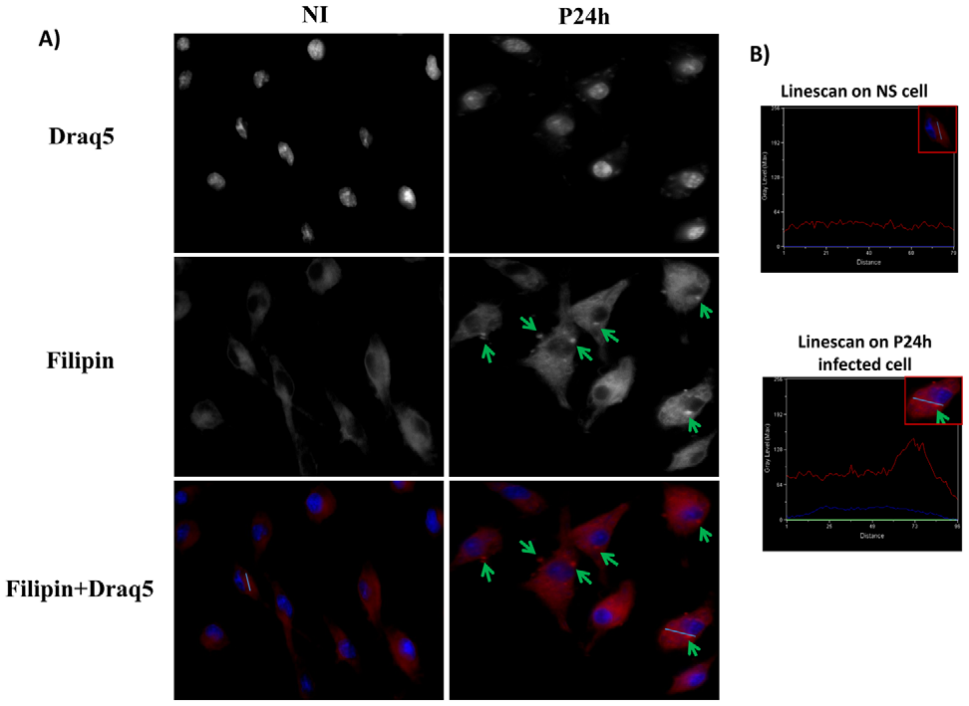
Free cholesterol accumulation in *Leishmania major* infected macrophages. BMdM were infected by *Leishmania major* promastigotes at different time points. Cells were fixed by formaldehyde, stained by Filipin for free cholesterol accumulation and visualized by Epifluorescence microscopy. The results are representative of two independent experiments. Only the results obtained 24 h post-infection is shown here. Similar results were observed for the other infection time point. B) Representative linescans (Metamorph software) of infected and non-infected cells that allow to quantify intensity differences in Filipin staining.

Macrophage cholesterol content is essentially a balance between cholesterol intake and/or biosynthesis and cholesterol efflux. Microarray data showed that transcription of two genes of the cholesterol biosynthesis pathway, HMG-CoA reductase and squalene epoxidase was increased by infection. A significant increase in the genes encoding different receptors implicated in the uptake of LDL was also observed. These include CD36 and the LDL receptor related protein, the low-density lipoprotein receptor-related protein 12 precursors (Lrp12). Infection also led to the expression of a variety of genes encoding secreted lipases such as the Lipoprotein lipase precursor (lpl) (1.7 fold) that preferentially hydrolyzes triglycerides. Finally, we observed a down-regulation of the genes encoding both the ATP-binding cassette transporter A1 (ABCA1) (almost 2 fold), which mediates the active efflux of cellular cholesterol and phospholipids, and the sterol 27-hydroxylase (CYP27) (2.7 fold) that plays an important role in oxysterol generation and thus in the prevention of cholesterol accumulation. Taken together, the transcriptomic data suggests an enhanced cholesterol uptake coupled with a decreased cholesterol efflux, raising the possibility that cholesterol accumulates within *L. major*-infected cells. To address this issue, macrophages were incubated with vehicule or infected with *Leishmania* promastigotes for the indicated period of time, fixed, and labeled with Filipin. Our data show that an intracellular Filipin labeling was detectable as early as one hpi. Progressively, the staining in the cytosol was enhanced and sustained for up to 24 hpi (Figure 4).

**Figure 5:**
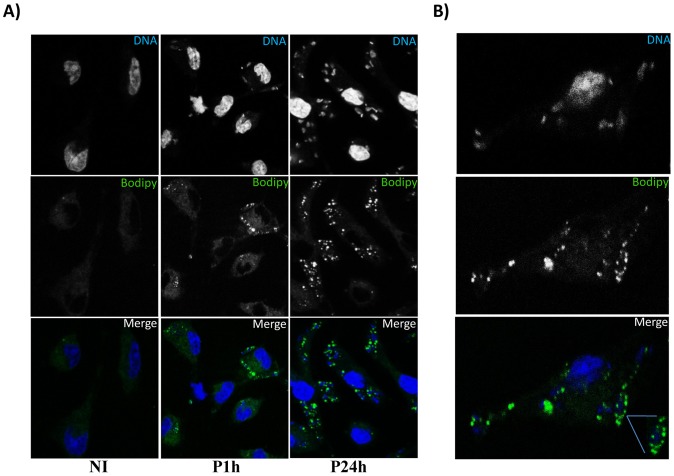
*Leishmania major* infection induces lipid droplet accumulation. BALB/c BMdM cells were infected by *Leishmania major* promastigotes at different time points. Cells were fixed by formaldehyde, stained by Bodipy493/503 for lipid droplet accumulation and visualized by confocal microscopy. A) Bodipy accumulation in *Leishmania* infected Macrophages. Only the results obtained one hour and twenty four hours after infection are shown here. Similar results were observed for the other infection time points. B) Lipid accumulation in close association with the parasitophorus vacuole. The results are representative of four independent experiments.

*Leishmania* infection also modulates the transcription of genes implicated in lipid metabolism. Indeed, expression of the genes encoding the stearoyl-CoA desaturases 2 (Scd), that catalyzes the conversion of stearoyl-CoA to oleoyl-CoA and thereby regulates the ratio of monounsaturated to saturated fatty acids was enhanced following *L. major* infection (up to 2.9) (Table S2). In addition to this important enzyme in the biosynthesis of fatty acids (FA), the mRNA levels for two other enzymes, the long chain fatty acid CoA ligase (Acsl1) (up to 2.47) (responsible for associating long-chain free fatty acids with coenzyme A) and the Fatty acid–binding protein 4 (FABP4) (up to 3.2), (that binds long-chain fatty acids and controls the cellular localisation of FAs) showed a sustained increase. Finally, transcription of the genes encoding three enzymes, namely the 1-acyl-sn-glycerol-3-phosphate acyltransferase 5 (Agapt5) that catalyses the formation of phosphatidic acid, the lipid phosphate phosphohydrolase 3 (Ppap2b), and the Diacylglycerol O-acyltransferase 2 (Dgat2), that catalyzes the conversion of diacylglycerol to triglycerides, was significantly enhanced in infected cells. As the formation of lipid droplets (LD) is highly linked to the biosynthesis of triglycerides, we investigated the role of infection on triglyceride accumulation and lipid-droplet formation in *Leishmania* infected BMdM. Using the fluorescent neutral lipid dye Bodipy, we observed a rapid (1 hour) accumulation of LD in *L. major*-infected cells (Figure 5A). This accumulation did not occur randomly, since in most cases, Bodipy-positives LD were present in close association with the parasitophorous vacuole (Figure 5B).

**Figure 6:**
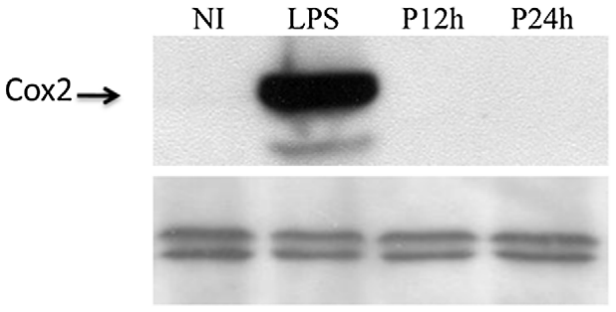
*Leishmania major* infection does not induce COX-2 protein production. Raw264.7 cells were either stimulated by LPS or infected by *L. major* promastigotes. Cells were then lysed at the indicated times, collected and finally subjected to Western blotting with antibodies specific for COX-2. Total ERK immunoblot was shown as loading control.

Expression of the gene encoding the cytosolic phospholipase A2 (cPLA2) was slightly upregulated (1.7 fold) while expression of the genes encoding the Prostaglandin G/H synthase 2 (COX-2) (up to 5.5) and the Prostaglandin E synthase displayed an important and sustained increase in response to *L. major* infection. By contrast, the gene encoding the Prostaglandin G/H synthase 1 (COX-1) was downregulated by more than two fold at 6 and 12 hpi. These findings suggest that *Leishmania* induces the biosynthetic pathway of the arachidonic acid (AA) cascade which involves the release of polyunsaturated fatty acids catalysed by the phosholipase A_2_ (PLA_2_) and its subsequent metabolism to bioactive prostaglandins (PGs) by cyclooxygenases. To investigate whether COX-2 mRNA expression correlated with protein synthesis, the protein levels of COX-2 were assessed in RAW 264.7 macrophages infected for 12 h and 24 h. LPS (100 ng/ml) was used as positive control. Figure 6 shows that, while LPS induced COX-2 expression, *L.* major promastigotes did not induce changes in the levels of COX-2.

#### Transcriptional profile induced by killed parasites

**Figure 7:**
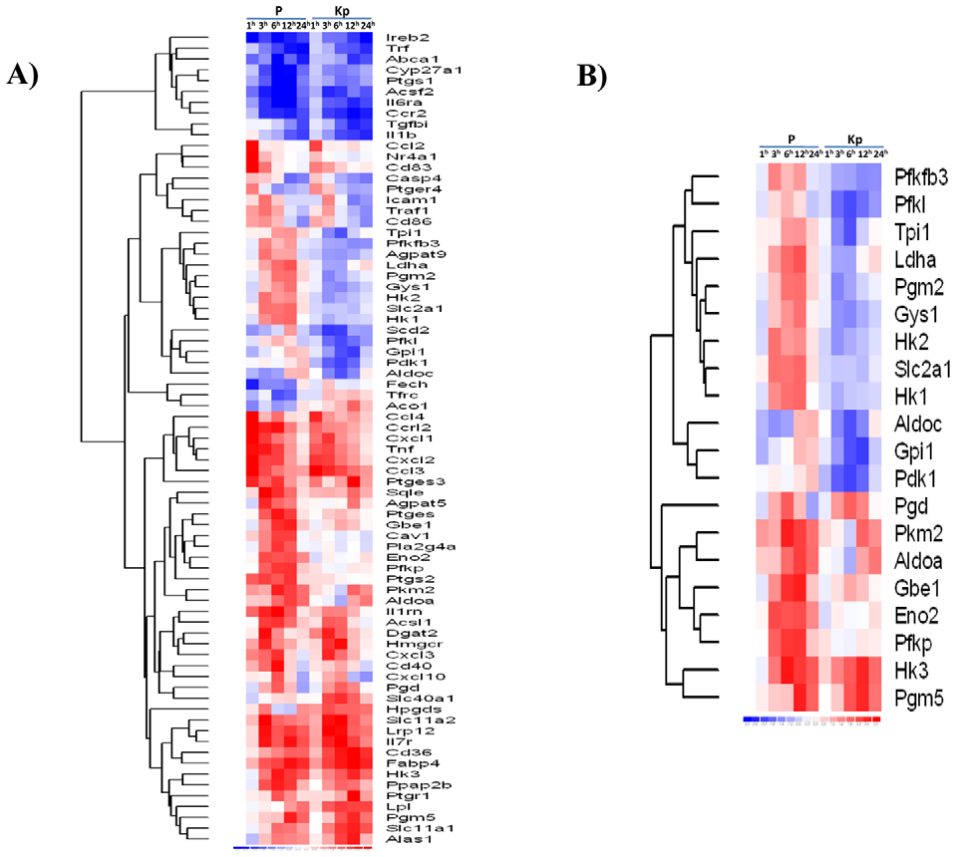
Hierarchic clustering of genes differentially regulated in macrophages following lived and heat-inactivated *Leishmania* promastigotes. The Heat Map show a partial list of genes classified through Ingenuity Pathways Analysis as (A) related to Cell Metabolic Pathways and significantly altered (p<0.05) in macrophages infected with live parasite (P) or heat-killed parasite (KP) versus non-infected macrophages across the time course of infection. (B) Implicated in carbohydrates between live and heat killed promastigotes. Red indicates gene induction; blue, gene repression.

In an attempt to highlight the cellular pathways actively regulated by the parasite, we used heat-inactivated *L. major* promastigotes as control. Compared to live promastigotes, killed parasites induced the expression of fewer genes. Only 375 genes were differentially expressed when macrophages internalized killed parasites. Of those, 241 genes were induced in both live and killed promastigote-infected BMdM. These include immune response genes such as chemokines, oxidative stress, but also metabolic pathway genes. The genes that were differentially regulated upon infection with *Leishmania* and which belong to the pathways analyzed upward, were subjected to hierarchical clustering analysis using DNA Chip Analyzer (dChip) software (Figure 7A and 7B). This clustering showed that infection of BMdM with either live or killed promastigotes resulted in similar transcriptomic profile for the genes implicated in the immune response and lipid metabolism. This result supports the hypothesis that the capacity to induce immune response, cholesterol accumulation and LD formation is independent of *Leishmania* viability. By contrast, internalization of heat-inactivated promastigotes clearly did not result in the expression of genes encoding glycolytic enzymes, suggesting that the activation and the shift to anaerobic glycolysis is actively regulated by the parasite (Figure 7B). These results have been confirmed by qPCR.

## Discussion

To identify host cell pathways modulated by *Leishmania*, DNA microarrays were used in several studies to monitor transcriptional changes in host cells following infection [1], [2], [4], [17]. As previously reported, we show here that *L. major* promastigotes induce the expression of genes encoding chemokines such as MIP-1alpha/CCL3, MIP-1beta/CCL4, potent chemotractants for monocytes/macrophages [2], and inflammatory mediators including TNFα, CCL5, CXCL1-3 [1]. We also observed an upregulation of CXCL10 gene expression, a Th1-mobilizing chemokine shown to be produced by lesion cells from self-healing cutaneous leishmaniasis (CL)-patients during active leishmaniasis [18], and the induction of the proinflammatory monocyte chemotractant protein 1 (MCP-1) encoded by the CCL2 gene, which has been associated to cutaneous leishmaniasis.

Besides inflammatory mediators, *L. major* promastigotes also induced the transcription of genes normally associated to an M2 response such as arginase1. A similar hybrid macrophage activation profile that does not strictly fall into one of the two categories (classical and alternative) has been previously observed in macrophages infected with *L. chagasi* [3]. This inflammatory response of infected macrophages is rapidly induced and is likely triggered by the stimulation of the receptors implicated in the recognition of *Leishmania* parasites, as heat-killed promastigotes displayed similar effect on macrophage mRNA profile.

Once the parasites get into the cells and start to multiply, the host cells seem to adapt their metabolism to face the infection. The microarray data indicated that *Leishmania* infection result in an enhanced rate of glycolysis but with reduced glucose flux through tricarboxylic acid cycle. The requirement to regenerate NAD^+^ to maintain glycolysis (i.e., conversion of glyceraldehyde-3-phosphate to 1,3-bisphosphoglycerate) is accomplished by lactate dehydrogenase (LDH)-catalyzed reduction of pyruvate to lactate. Thus, infected macrophages would tend to convert glucose into lactate even in the presence of sufficient oxygen to support mitochondrial oxidative phosphorylation. Moreover, the down-regulation of a number of genes implicated in the TCA cycle and oxidative phosphorylation suggests that increased glycolysis may be the mechanism *L. major* relies on for energy production. Interestingly, expression of the glycolytic enzymes encoding genes, including LDH and PDK, was not induced when heat-killed promastigotes were used to infect BMdM, suggesting that *L. major* promastigotes actively induce macrophages to shift their metabolism to anaerobic glycolysis. Up-regulation of host glycolytic transcripts has been reported for macrophages infected by *Toxoplasma gondii* [19] and was shown to correlate with the activation of the HIF-1α transcription factor [20]. On the other hand, expression of HIF-1α has been observed in the cutaneous lesions of *L. amazonensis* infected BALB/c mice [21]. Stabilization of HIF1α in response to *Leishmania* infection may explain this increasing flux through the glycolytic pathway, the conversion of pyruvate to lactate and the suppression of the TCA cycle, and the oxidative phosphorylation observed in infected BMdM. Whether the effect of *Leishmania* on this metabolic pathway relies on HIF-1α activation is currently under investigation.

Almost all the scavenger receptors such as CD36, expressed on several cell types including macrophages, deliver associated lipids to lysosomes. Then, essential enzymes for the cleavage of cholesteryl esters and triglycerides generate free cholesterol and fatty acids that are next released from lysosomes into the cytosol. The efflux of excess cholesterol is promoted by different transporters including the ATP binding cassette (ABC) gene family. Among those, ABCA1 plays a major role in this process and decreased expression of this transporter, as found in our microarray data, could lead to the inhibition of the cholesterol efflux. Moreover, our data indicate a down-regulation of CYP27 mRNA levels in *Leishmania*-infected cells, suggesting that infection results in an alteration of the host cell oxysterol content. Decreased levels of this natural Liver X Receptor (LXR) endogenous ligand in infected cells may explain the inhibition of LXR target genes expression such ABCA1 [22], [23]. Moreover, stearoyl-CoA desaturase, whose expression was induced by *Leishmania*, inhibits ABCA1-mediated cholesterol efflux [24]. Epifluorescence microscopy confirmed the microarray data, indicating that *L. major* infection modulates macrophage cholesterol content and induces its accumulation into the cytoplasm. Cholesterol plays a key role during the infection process by several intracellular pathogens, including adhesion and internalization [25], as well as for survival within host cells as reported for the protozoan parasite *Toxoplasma gondii* [26], [27]. In *Leishmania*-infected cells, cholesterol depletion [28] or sequestration [29] reduces the extent of infection by promastigotes. The role of cholesterol accumulation into *L. major*-infected cells remains to be defined and whether this accumulation is induced by other *Leishmania* species remains to be investigated.

*L. major* infection also promotes macrophage-derived foam cell formation. This was suggested by the analysis of microarray data and was validated by the staining of infected cells with Bodipy that clearly demonstrated the accumulation of lipids droplets into the cytoplasm. Interestingly, these lipid droplets are mainly localized in the proximity of the parasitophorous vacuoles. The induction of foam macrophages has been observed during infection by different bacteria such as *Mycobacterium*, *Chlamydia* [30] or parasites including *Trypanosoma cruzi* [31], *Toxoplasma gondii* [32], [33], and *Plasmodium falciparum* [34]. Moreover, a recent study revealed that cytoplasmic LDs are translocated across the inclusion membrane into the lumen of the parasitophorous vacuole (PV) in *Chlamydia* infected cells [35]. A similar mechanism of lipid acquisition by *Leishmania* may exist that allows the parasite to take advantage of this high-energy source. From another point of view, accumulation of LD may be implicated in the cross-presentation of antigens derived from PVs containing live parasites. Indeed, accumulation of lipid bodies has been reported to be required for normal and efficient cross-presentation of OVA-coated latex beads by dendritic cells [36]. Interestingly, expression of a member of the IRG gene family (Irgm3) that has recently been implicated in this pathway was induced in *Leishmania*-infected BMdM. Both live and heat-inactivated *Leishmania* promastigotes induce the transcription of the same set of genes involved in the intracellular cholesterol accumulation and foam cell formation in bone marrow-derived macrophages suggesting that the development of the parasite is not required. Accumulation of cholesterol and formation of lipids bodies may be achieved through the stimulation of different receptors such as Toll-like receptors (TLRs) that have been implicated in the recognition and control of *Leishmania* parasites [37], [38], [39]. Indeed, a recent study revealed that TLR stimulation impairs macrophage cellular cholesterol efflux *in vivo* [40]. Moreover, *in vitro* activation of TLR-3 and TLR-4 by microbial ligand blocks the induction of LXR target genes including ABCA1 [41]. Similarly, the formation of lipid bodies in response to bacteria is dependent on TLR (and particularly TLR2) signalling [42] and more generally the activation of TLRs by pathogen-derived agonists promotes lipid accumulation [43], [44]. TLR may thus provide an important link between lipid metabolism, infection and the innate immune response.

In inflammatory cells, lipid bodies have been associated to arachidonic acid metabolism and eicosanoid-forming enzymes have been localized in lipid bodies that are sites for 5-LO- and COX-derived eicosanoid synthesis [45]. Moreover, lipid bodies have been reported as intracellular domains for eicosanoid synthesis *in vivo*. Consistent with previous studies [17], [46], we show here that despite the induction of significant COX-2 mRNA levels the COX-2 protein was not detected in *L. major* infected macrophages. The control of gene expression in eucaryotes is subjected to dynamic regulation in the cell. This control is a multi-step process that includes transcription, splicing, translation and post-translational regulation. We have thus to take in mind that besides transcription, different other levels of control, may take place in *L. major*-infected macrophages and influence the level of biologically active protein.

Collectively, our results suggest that *L. major* promastigotes push the macrophages to shift toward anaerobic glycolysis and induce the accumulation of cholesterol and the formation of foam cells. These metabolic changes occurring in host cells appear to be induced by a large number of pathogens, and are likely to play an important role in pathogenesis.

## Supporting Information

Table S1 **Canonical pathways identified by Inguenuity Pathway Analysis software as significantly altered (p<0.05) in** ***L. major*** **infected BALB/c macrophages.**
(PDF)

Table S2 **Selected genes up- or down-regulated more than two-fold in** ***Leishmania*** **infected BMdM as measured by array data.** Results are expressed as Log_2_ of the fold change (Log_2_(FC)). The numbers presented for each time point are the average of the three biological replicates. When the modulation of mRNA level is not statistically significant, the Log_2_(FC) was denoted 0.
(PDF)

Table S3 **Selected genes up- or down-regulated more than two-fold in** ***Leishmania*** **infected BMdM as measured by qRT-PCR.** Genes selected in Table S2 were tested by qRT-PCR. Changes in mRNA levels are calculated using the 2^−ΔΔ*CT*^ method. The numbers presented for each time point are the average of the three biological replicates.
(PDF)
