# Supplementary material for: Transcriptomic Signature of Leishmania Infected Mice Macrophages: A Metabolic Point of View
Source: PLoS Negl Trop Dis. 2012 Aug 21;6(8):e1763. doi: 10.1371/journal.pntd.0001763 (PMC3424254; doi:10.1371/journal.pntd.0001763)
Supplement: Table S1 — Canonical pathways identified by Inguenuity Pathway Analysis software as significantly altered (p<0.05) in L. major infected BALB/c macrophages. (PDF) [file pntd.0001763.s001.pdf]

# Table S1 : Canonical pathways identified by Inguenuity Pathway Analysis™ software as significantly altered in *L. major* infected BALB/c macrophages

**© 2000-2010 Ingenuity Systems, Inc. All rights reserved.**

| Ingenuity Canonical Pathways | AnalysisName | -log(p-value) | Ratio | Molecules |
|---|---|---|---|---|
| Glycolysis/Gluconeogenesis | 3h | 5,4E00 | 7,04E-02 | HK1, HK2, ALDH3A2, PGM1, ENO2, HK3, PFKP, PFKL, ACSL1, LDHA |
| Glycolysis/Gluconeogenesis | 6h | 8,3E00 | 9,86E-02 | PKM2, PGM1, ENO2, PFKP, PFKL, TPI1, HK1, HK2, ALDH3A2, ALDOA, HK3, LDHA, PTGR1, ACSL1 |
| Glycolysis/Gluconeogenesis | 12h | 9,71E00 | 9,86E-02 | PGM1, ENO2, PFKP, PFKL, TPI1, HK1, GPI, HK2, PGM5, ALDOA, HK3, ACSL1, LDHA, ALDOC |
| Glycolysis/Gluconeogenesis | 24h | 1,75E00 | 1,41E-02 | ENO2, HK3 |
| Galactose Metabolism | 3h | 3,73E00 | 5,22E-02 | HK1, HK2, PGM1, HK3, PFKP, PFKL |
| Galactose Metabolism | 6h | 5,2E00 | 6,96E-02 | HK1, GLA, HK2, AKR1B10, PGM1, HK3, PFKP, PFKL |
| Galactose Metabolism | 12h | 4,93E00 | 6,09E-02 | HK1, HK2, PGM5, PGM1, HK3, PFKP, PFKL |
| Galactose Metabolism | 24h | 9,82E-01 | 8,7E-03 | HK3 |
| Fructose and Mannose Metabolism | 3h | 3,38E00 | 4,14E-02 | PFKFB3, HK1, HK2, HK3, PFKP, PFKL |
| Fructose and Mannose Metabolism | 6h | 5,68E00 | 6,21E-02 | PFKFB3, HK1, HK2, AKR1B10, ALDOA, HK3, PFKP, PFKL, TPI1 |
| Fructose and Mannose Metabolism | 12h | 6,59E00 | 6,21E-02 | PFKFB3, HK1, HK2, ALDOA, HK3, PFKP, PFKL, TPI1, ALDOC |
| Fructose and Mannose Metabolism | 24h | 9,23E-01 | 6,9E-03 | HK3 |
| Arginine and Proline Metabolism | 3h | 2,6E00 | 3,28E-02 | P4HA1, RARS, ALDH3A2, SMOX, GATM, P4HA2 |
| Arginine and Proline Metabolism | 6h | 2,23E00 | 3,28E-02 | P4HA1, ALDH3A2, SMOX, NOS2, GATM, P4HA2 |
| Arginine and Proline Metabolism | 12h | 3,55E00 | 3,83E-02 | P4HA1, P4HB, GOT1, SMOX, NOS2, GATM, P4HA2 |
| Arginine and Proline Metabolism | 24h | 7,86E-01 | 5,46E-03 | P4HA2 |
| Glutathione Metabolism | 3h | 2,47E00 | 5,1E-02 | GSR, LNPEP, GCLC, GCLM, IDH1 |
| Glutathione Metabolism | 6h | 4,6E00 | 8,16E-02 | GSR, PGD, GSTM5, GCLC, GCLM, GLRX, RNPEP, IDH1 |
| Glutathione Metabolism | 12h | 4,39E00 | 7,14E-02 | GSR, GSTM5, HPGDS, GCLM, GLRX, RNPEP, IDH1 |
| Glutathione Metabolism | 24h | 9,07E-01 | 1,02E-02 | GSTM5 |
| Glutamate Metabolism | 3h | 2,34E00 | 5,13E-02 | GSR, GLUL, GCLC, GCLM |
| Glutamate Metabolism | 6h | 2,08E00 | 5,13E-02 | GSR, GLUL, GCLC, GCLM |
| Glutamate Metabolism | 12h | 3,42E00 | 6,41E-02 | GSR, GFPT1, GLUL, GOT1, GCLM |
| Starch and Sucrose Metabolism | 3h | 2,21E00 | 3,05E-02 | HK1, GYS1, HK2, PGM1, GBE1, HK3 |
| Starch and Sucrose Metabolism | 6h | 2,58E00 | 3,55E-02 | HK1, UGDH, GYS1, HK2, PGM1, GBE1, HK3 |
| Starch and Sucrose Metabolism | 12h | 4,93E00 | 4,57E-02 | HK1, GPI, UGDH, GYS1, HK2, PGM5, PGM1, GBE1, HK3 |
| Starch and Sucrose Metabolism | 24h | 2E00 | 1,02E-02 | GBE1, HK3 |
| Nicotinate and Nicotinamide Metabolism | 3h | 1,96E00 | 4,41E-02 | PIM1, CDK6, MAPK6, NAMPT, MAP2K3, MAP2K1 |
| Nicotinate and Nicotinamide Metabolism | 6h | 7,39E-01 | 2,94E-02 | PAK1, CDK6, NADK (includes EG:65220), MAP2K1 |
| Nicotinate and Nicotinamide Metabolism | 12h | 2,11E00 | 4,41E-02 | CDK6, MAPK6, NAMPT, NADK (includes EG:65220), MAP2K1, BST1 |
| Methionine Metabolism | 3h | 1,81E00 | 3,75E-02 | DNMT3A, MTR, MAT2A |
| Methionine Metabolism | 6h | 3,28E-01 | 1,25E-02 | MTR |
| Methionine Metabolism | 12h | 4,1E-01 | 1,25E-02 | MTR |
| Selenoamino Acid Metabolism | 3h | 1,62E00 | 3,9E-02 | SEPHS2, METTL6, MAT2A |
| Selenoamino Acid Metabolism | 6h | 2,78E-01 | 1,3E-02 | SEPHS2 |
| Selenoamino Acid Metabolism | 12h | 3,56E-01 | 1,3E-02 | SEPHS2 |
| Pentose Phosphate Pathway | 3h | 1,56E00 | 3,37E-02 | PGM1, PFKP, PFKL |
| Pentose Phosphate Pathway | 6h | 2,99E00 | 5,62E-02 | PGD, PGM1, ALDOA, PFKP, PFKL |
| Pentose Phosphate Pathway | 12h | 5,69E00 | 7,87E-02 | GPI, PGM5, PGM1, ALDOA, PFKP, PFKL, ALDOC |
| Inositol Phosphate Metabolism | 3h | 1,45E00 | 3,41E-02 | PIM1, CDK6, MAPK6, MAP2K3, PIK3CD, MAP2K1 |
| Inositol Phosphate Metabolism | 6h | 3,18E00 | 5,68E-02 | PLCB2, PAK1, PLCG2, PIKFYVE, CDK6, PIK3CD, INPP5K, PIP4K2A, MAP2K1, INPP5D |
| Inositol Phosphate Metabolism | 12h | 1,12E00 | 2,84E-02 | CDK6, MAPK6, IMPA2, PIK3CB, MAP2K1 |
| One Carbon Pool by Folate | 3h | 1,35E00 | 5,13E-02 | MTR, MTHFD1L |
| One Carbon Pool by Folate | 6h | 2,15E00 | 7,69E-02 | EHD4, MTR, MTHFD1L |
| One Carbon Pool by Folate | 12h | 1,41E00 | 5,13E-02 | MTR, MTHFD1L |
| Inositol Metabolism | 3h | 1,18E00 | 4,08E-02 | POR, HMOX1, ERO1L, DEGS1 |
| Inositol Metabolism | 6h | 2,01E00 | 6,12E-02 | HMOX1, CXORF21, ALDOA, ERO1L, TPI1, FADS1 |
| Inositol Metabolism | 12h | 3,27E00 | 7,14E-02 | HMOX1, ALDOA, ERO1L, TPI1, FADS1, FZD7, ALDOC |
| Histidine Metabolism | 3h | 1,15E00 | 2,5E-02 | ALDH3A2, METTL6, SMOX |
| Histidine Metabolism | 6h | 4,98E-01 | 1,67E-02 | ALDH3A2, SMOX |
| Histidine Metabolism | 12h | 2,27E-01 | 8,33E-03 | SMOX |
| N-Glycan Degradation | 3h | 1,01E00 | 6,25E-02 | MAN2A2, ENGASE |
| N-Glycan Degradation | 6h | 2,48E00 | 1,25E-01 | MAN1C1, MAN2B1, MAN2A2, ENGASE |
| N-Glycan Degradation | 12h | 2,87E00 | 1,25E-01 | MAN1C1, MAN2B1, MAN2A2, ENGASE |
| O-Glycan Biosynthesis | 3h | 9,62E-01 | 4,26E-02 | GCNT1, ST3GAL1 |
| O-Glycan Biosynthesis | 6h | 3,06E-01 | 2,13E-02 | GCNT1 |
| Propanoate Metabolism | 3h | 9,52E-01 | 2,31E-02 | ALDH3A2, ACSL1, LDHA |
| Propanoate Metabolism | 6h | 7,9E-01 | 2,31E-02 | ALDH3A2, ACSL1, LDHA |
| Propanoate Metabolism | 12h | 5,31E-01 | 1,54E-02 | ACSL1, LDHA |
| Aminosugars Metabolism | 3h | 9,07E-01 | 2,46E-02 | HK1, HK2, HK3 |
| Aminosugars Metabolism | 6h | 7,48E-01 | 2,46E-02 | HK1, HK2, HK3 |
| Aminosugars Metabolism | 12h | 3,87E00 | 5,74E-02 | HK1, GFPT1, HK2, PDE3B, PDE1B, CYB5R3, HK3 |
| Aminosugars Metabolism | 24h | 8,32E-01 | 8,2E-03 | HK3 |
| Lysine Degradation | 3h | 8,84E-01 | 2,14E-02 | CNDP2, ALDH3A2, DOT1L, MMP13, CASP4 |
| Lysine Degradation | 6h | 4,04E-01 | 1,71E-02 | CNDP2, ALDH3A2, DOT1L, MMP13 |
| Lysine Degradation | 12h | 3,45E-01 | 1,28E-02 | PLOD2, DOT1L, MMP13 |
| Lysine Degradation | 24h | 5,31E-01 | 4,27E-03 | DOT1L |
| Pyruvate Metabolism | 3h | 8,25E-01 | 2,01E-02 | ALDH3A2, ACSL1, LDHA |
| Pyruvate Metabolism | 6h | 2,29E00 | 4,03E-02 | GRHPR, PKM2, ALDH3A2, AKR1B10, ACSL1, LDHA |
| Pyruvate Metabolism | 12h | 8,96E-01 | 2,01E-02 | HAGH, ACSL1, LDHA |
| Glycosphingolipid Biosynthesis - Lactoseries | 3h | 8,03E-01 | 3,7E-02 | ST3GAL1 |
| Bile Acid Biosynthesis | 3h | 7,61E-01 | 2E-02 | CYP27A1, ALDH3A2 |
| Bile Acid Biosynthesis | 6h | 1,22E00 | 3E-02 | CYP27A1, ALDH3A2, PTGR1 |
| Bile Acid Biosynthesis | 12h | 2,96E-01 | 1E-02 | CYP27A1 |
| Nitrogen Metabolism | 3h | 7,28E-01 | 1,5E-02 | GLUL, ASNS |
| Nitrogen Metabolism | 6h | 6,15E-01 | 1,5E-02 | GLUL, ASNS |
| Nitrogen Metabolism | 12h | 2,82E-01 | 7,52E-03 | GLUL |
| Tryptophan Metabolism | 3h | 7,03E-01 | 1,57E-02 | ALDH3A2, METTL6, SMOX, PTGS2 |
| Tryptophan Metabolism | 6h | 2,86E-01 | 1,18E-02 | ALDH3A2, SMOX, PTGS2 |
| Tryptophan Metabolism | 12h | 4,46E-01 | 1,18E-02 | CAT, SMOX, PTGS2 |
| Keratan Sulfate Biosynthesis | 3h | 6,82E-01 | 3,64E-02 | ST3GAL1, B4GALT5 |
| Keratan Sulfate Biosynthesis | 12h | 2,62E-01 | 1,82E-02 | WDFY3 |
| Keratan Sulfate Biosynthesis | 24h | 9,82E-01 | 1,82E-02 | WDFY3 |
| Glycerolipid Metabolism | 3h | 6,28E-01 | 1,92E-02 | AGPAT5, ALDH3A2, PPAP2B |
| Glycerolipid Metabolism | 6h | 1,82E00 | 3,85E-02 | GLA, ALDH3A2, PPAP2B, AKR1B10, PNPLA2, PTGR1 |
| Glycerolipid Metabolism | 12h | 3,36E-01 | 1,28E-02 | PPAP2B, APOC2 |
| Folate Biosynthesis | 3h | 6,05E-01 | 1,25E-02 | GCH1 |
| Folate Biosynthesis | 6h | 5,4E-01 | 1,25E-02 | GCH1 |
| N-Glycan Biosynthesis | 3h | 6,03E-01 | 2,15E-02 | MAN2A2, B4GALT5 |
| N-Glycan Biosynthesis | 6h | 9,74E-01 | 3,23E-02 | MAN1C1, WDFY3, MAN2A2 |
| N-Glycan Biosynthesis | 12h | 1,23E00 | 3,23E-02 | MAN1C1, WDFY3, MAN2A2 |
| N-Glycan Biosynthesis | 24h | 9,31E-01 | 1,08E-02 | WDFY3 |
| Glyoxylate and Dicarboxylate Metabolism | 3h | 5,59E-01 | 8,55E-03 | MTHFD1L |
| Glyoxylate and Dicarboxylate Metabolism | 6h | 1,26E00 | 1,71E-02 | GRHPR, MTHFD1L |
| Glyoxylate and Dicarboxylate Metabolism | 12h | 5,87E-01 | 8,55E-03 | MTHFD1L |
| Phenylalanine, Tyrosine and Tryptophan Biosynthesis | 3h | 5,38E-01 | 1,49E-02 | ENO2 |
| Phenylalanine, Tyrosine and Tryptophan Biosynthesis | 6h | 4,75E-01 | 1,49E-02 | ENO2 |
| Phenylalanine, Tyrosine and Tryptophan Biosynthesis | 12h | 1,41E00 | 2,99E-02 | ENO2, GOT1 |
| Phenylalanine, Tyrosine and Tryptophan Biosynthesis | 24h | 1,37E00 | 1,49E-02 | ENO2 |
| Tyrosine Metabolism | 3h | 4,68E-01 | 9,9E-03 | METTL6, SMOX |
| Tyrosine Metabolism | 6h | 3,75E-01 | 9,9E-03 | SMOX, PTGR1 |
| Tyrosine Metabolism | 12h | 5,13E-01 | 9,9E-03 | GOT1, SMOX |
| Glycine, Serine and Threonine Metabolism | 3h | 4,43E-01 | 1,33E-02 | SMOX, GATM |
| Glycine, Serine and Threonine Metabolism | 6h | 1,77E00 | 3,33E-02 | GRHPR, PLCB2, PLCG2, SMOX, GATM |
| Glycine, Serine and Threonine Metabolism | 12h | 9,51E-01 | 2E-02 | GOT1, SMOX, GATM |
| Glycosphingolipid Biosynthesis - Globoseries | 3h | 4,22E-01 | 2,17E-02 | ST3GAL1 |
| Glycosphingolipid Biosynthesis - Globoseries | 6h | 3,64E-01 | 2,17E-02 | GLA |
| Ascorbate and Aldarate Metabolism | 3h | 4,22E-01 | 1,16E-02 | ALDH3A2 |
| Ascorbate and Aldarate Metabolism | 6h | 3,64E-01 | 1,16E-02 | ALDH3A2 |
| Glycerophospholipid Metabolism | 3h | 4,04E-01 | 1,55E-02 | HMOX1, AGPAT5, PPAP2B |
| Glycerophospholipid Metabolism | 6h | 1,27E00 | 3,11E-02 | HMOX1, PLA2G4A, PLCB2, LPCAT2, PLCG2, PPAP2B |
| Glycerophospholipid Metabolism | 12h | 1,23E00 | 2,59E-02 | HMOX1, PLA2G4A, LPCAT2, PPAP2B, GOT1 |
| Glycerophospholipid Metabolism | 24h | 5,94E-01 | 5,18E-03 | LPCAT2 |
| Aminophosphonate Metabolism | 3h | 3,84E-01 | 1,54E-02 | METTL6 |
| Glycosphingolipid Biosynthesis - Ganglioseries | 3h | 3,73E-01 | 1,54E-02 | ST3GAL1 |
| Citrate Cycle | 3h | 3,62E-01 | 1,69E-02 | IDH1 |
| Citrate Cycle | 6h | 3,06E-01 | 1,69E-02 | IDH1 |
| Citrate Cycle | 12h | 1,02E00 | 3,39E-02 | IDH3G, IDH1 |
| Biosynthesis of Steroids | 3h | 3,62E-01 | 7,81E-03 | SQLE |
| Biosynthesis of Steroids | 12h | 3,87E-01 | 7,81E-03 | EBP |
| Urea Cycle and Metabolism of Amino Groups | 3h | 3,51E-01 | 1,25E-02 | GATM |
| Urea Cycle and Metabolism of Amino Groups | 6h | 2,97E-01 | 1,25E-02 | GATM |
| Urea Cycle and Metabolism of Amino Groups | 12h | 9,94E-01 | 2,5E-02 | CAT, GATM |
| Phospholipid Degradation | 3h | 3,38E-01 | 1,89E-02 | HMOX1, PPAP2B |
| Phospholipid Degradation | 6h | 1,43E00 | 4,72E-02 | HMOX1, PLA2G4A, PLCB2, PLCG2, PPAP2B |
| Phospholipid Degradation | 12h | 7,64E-01 | 2,83E-02 | HMOX1, PLA2G4A, PPAP2B |
| Phenylalanine Metabolism | 3h | 3,31E-01 | 9,17E-03 | SMOX |
| Phenylalanine Metabolism | 6h | 7,73E-01 | 1,83E-02 | PRDX1, SMOX |
| Phenylalanine Metabolism | 12h | 9,49E-01 | 1,83E-02 | GOT1, SMOX |
| Glycosaminoglycan Degradation | 3h | 3,22E-01 | 2,78E-02 | SGSH, HYAL1 |
| Glycosaminoglycan Degradation | 6h | 7,53E-01 | 4,17E-02 | SGSH, HYAL1, SULF2 |
| Aminoacyl-tRNA Biosynthesis | 3h | 2,96E-01 | 1,2E-02 | RARS |
| Alanine and Aspartate Metabolism | 3h | 2,73E-01 | 1,14E-02 | ASNS |
| Alanine and Aspartate Metabolism | 6h | 2,24E-01 | 1,14E-02 | ASNS |
| Alanine and Aspartate Metabolism | 12h | 8,13E-01 | 2,27E-02 | ADSSL1, GOT1 |
| Chondroitin Sulfate Biosynthesis | 3h | 2,46E-01 | 1,49E-02 | CHSY1 |
| Chondroitin Sulfate Biosynthesis | 6h | 2E-01 | 1,49E-02 | DSE |
| Cysteine Metabolism | 3h | 2,4E-01 | 1,11E-02 | LDHA |
| Cysteine Metabolism | 12h | 7,33E-01 | 2,22E-02 | GOT1, LDHA |
| Fatty Acid Metabolism | 3h | 2,28E-01 | 1,04E-02 | ALDH3A2, ACSL1 |
| Fatty Acid Metabolism | 6h | 3,77E-01 | 1,56E-02 | ALDH3A2, PTGR1, ACSL1 |
| β-alanine Metabolism | 3h | 2,17E-01 | 1,02E-02 | ALDH3A2 |
| Arachidonic Acid Metabolism | 6h | 8,88E-01 | 2,2E-02 | PLA2G4A, PTGES, PTGES3 (includes EG:10728), PTGS1, PTGS2 |
| Arachidonic Acid Metabolism | 12h | 1,24E00 | 2,2E-02 | PLA2G4A, PTGES, PTGS1, HPGDS, PTGS2 |
| Linoleic Acid Metabolism | 6h | 2,86E-01 | 1,61E-02 | PLA2G4A, FADS1 |
| Linoleic Acid Metabolism | 12h | 4,1E-01 | 1,61E-02 | PLA2G4A, FADS1 |
| Sphingolipid Metabolism | 6h | 1,07E00 | 3,57E-02 | GLA, PPAP2B, SGPP1, SULF2 |
| Oxidative Phosphorylation | 6h | 2,77E-01 | 1,8E-02 | ATP6V1E1, ATP6V1H, ATP6V1C1 |
| Purine Metabolism | 6h | 5,14E-01 | 1,82E-02 | PKM2, RP2, ENTPD1, ABCC1, ATF7IP, AK3L1, CASK, ATP13A2 |
| Purine Metabolism | 12h | 4,25E-01 | 1,37E-02 | ADSSL1, ENTPD1, PDE3B, ABCC1, PDE1B, IFNAR1 |
| Purine Metabolism | 24h | 2,98E-01 | 2,28E-03 | ENTPD1 |
| Pyrimidine Metabolism | 6h | 1,03E00 | 2,6E-02 | RP2, ENTPD1, EHD4, AK3L1, TXN, TXNRD1 |
| Pyrimidine Metabolism | 24h | 5,39E-01 | 4,33E-03 | ENTPD1 |
| Nucleotide Sugars Metabolism | 6h | 5,65E-01 | 1,35E-02 | UGDH |
| Nucleotide Sugars Metabolism | 12h | 6,61E-01 | 1,35E-02 | UGDH |
| Metabolism of Xenobiotics by Cytochrome P450 | 6h | 2,53E-01 | 9,57E-03 | GSTM5, PTGR1 |
| Metabolism of Xenobiotics by Cytochrome P450 | 12h | 3,71E-01 | 9,57E-03 | GSTM5, HPGDS |
| Metabolism of Xenobiotics by Cytochrome P450 | 24h | 7,35E-01 | 4,78E-03 | GSTM5 |
| Methane Metabolism | 6h | 5,4E-01 | 1,52E-02 | PRDX1 |
| Methane Metabolism | 12h | 6,35E-01 | 1,52E-02 | CAT |
| Riboflavin Metabolism | 6h | 9,99E-01 | 3,64E-02 | ACP5, SGPP1 |
| C21-Steroid Hormone Metabolism | 12h | 5,28E-01 | 1,41E-02 | EBP |
| Pentose and Glucuronate Interconversions | 6h | 8,39E-01 | 1,32E-02 | UGDH, AKR1B10 |
| Pentose and Glucuronate Interconversions | 12h | 3,87E-01 | 6,62E-03 | UGDH |
| Stilbene, Coumarine and Lignin Biosynthesis | 6h | 4,06E-01 | 1,28E-02 | PRDX1 |
