# Supplementary material for: Transcriptomic Signature of Leishmania Infected Mice Macrophages: A Metabolic Point of View
Source: PLoS Negl Trop Dis. 2012 Aug 21;6(8):e1763. doi: 10.1371/journal.pntd.0001763 (PMC3424254; doi:10.1371/journal.pntd.0001763)
Supplement: Table S2 — Selected genes up- or down-regulated more than two-fold in Leishmania infected BMdM as measured by array data. Results are expressed as Log2 of the fold change (Log2(FC)). The numbers presented for each time point are the average of the three biological replicates. When the modulation of mRNA level is not statistically significant, the Log2(FC) was denoted 0. (PDF) [file pntd.0001763.s002.pdf]

Table S2 : Selected genes up- or down-regulated more than two-fold in Leishmania infected BMdM as measured by array data

Ensembl.Gene.ID	Description	MGI Symbol	Pathways	Log2(FC)-1h	Log2 (Fc)-3h	Log2 (Fc)-6h	Log2 (Fc)-12h	Log2 (Fc)-24h
ENSMUSG00000028645	Solute carrier family 2, facilitated glucose transporter member 1 (Glucose transporter type 1, e	Slc2a1	Glycolysis, pathway	0	3,214378018	2,42716028	2,581617141	0
ENSMUSG00000036427	Glucose-6-phosphate isomerase (EC 5.3.1.9) (GPI) (Phosphoglucose isomerase) (PGI) (Phospho	Gpi1		0	0	0	1,337895056	0
ENSMUSG00000020277	6-phosphofructokinase, liver type (EC 2.7.1.11) (Phosphofructokinase 1) (Phosphohexokinase)	Pfkl		0	1,214591183	0,95199684	0,766093313	0
ENSMUSG00000021196	6-phosphofructokinase type C (EC 2.7.1.11) (Phosphofructokinase 1) (Phosphohexokinase) (Ph	Pfkp		0	1,33842839	1,77882142	1,718663934	0
ENSMUSG00000030695	aldolase 1, A isoform, retrogene 1 [Source:MGI;Acc:MGI:2447811]	Aldoa		0	0	0,81391449	0,920161	0
ENSMUSG00000017390	Fructose-bisphosphate aldolase C (EC 4.1.2.13) (Brain-type aldolase) (Aldolase 3) (Zebrin II) (Sc	Aldoc		0	0	0	0,791231297	0
ENSMUSG00000023456	Triosephosphate isomerase (EC 5.3.1.1) (TIM) (Triose-phosphate isomerase). [Source:Uniprot/	Tpi1		0	0	1,85248647	1,698747114	0
ENSMUSG00000004267	Gamma-enolase (EC 4.2.1.11) (2-phospho-D-glycerate hydro-lyase) (Neural enolase) (Neuron-s	Eno2		0	1,68801578	1,68675772	1,719062067	1,36955953
ENSMUSG00000032294	Pyruvate kinase isozymes M1/M2 (EC 2.7.1.40) (Pyruvate kinase muscle isozyme). [Source:Uni	Pkm2		0	0	1,05244608	0	0
ENSMUSG00000057666	Glyceraldehyde-3-phosphate dehydrogenase (EC 1.2.1.12) (GAPDH). [Source:Uniprot/SWISSPR	Gapdh		0	0	0	0,926	0
ENSMUSG00000026773	6-phosphofructo-2-kinase/fructose-2,6-biphosphatase 3 [Source:RefSeq_peptide;Acc:NP_5734	Pfkfb3		0	1,530869524	0,9722233	1,349865181	0
ENSMUSG00000006494	Pyruvate dehydrogenase [lipoamide]] kinase isozyme 1, mitochondrial precursor (EC 2.7.11.2)	Pdk1		0	0,712072524	0,75918556	0	0
ENSMUSG00000022707	1,4-alpha-glucan-branching enzyme (EC 2.4.1.18) (Glycogen-branching enzyme) (Brancher enzy	Gbe1	strach degradation	0	1,208213876	2,03964157	2,218365553	1,133666409
ENSMUSG00000025791	Phosphoglucomutase-1 (EC 5.4.2.2) (Glucose phosphomutase 1) (PGM 1). [Source:Uniprot/SW	Pgm2		0	1,514197843	1,86392124	1,697257073	0
ENSMUSG00000041731	Phosphoglucomutase-like protein 5. [Source:Uniprot/SWISSPROT;Acc:Q8BZF8]	Pgm5		0	0	0	0,735801575	0
ENSMUSG00000037012	Hexokinase-1 (EC 2.7.1.1) (Hexokinase type I) (HK I) (Hexokinase, tumor isozyme). [Source:Uni	Hk1		0	1,206750136	1,12417842	1,277000206	0
ENSMUSG00000000628	Hexokinase-2 (EC 2.7.1.1) (Hexokinase type II) (HK II). [Source:Uniprot/SWISSPROT;Acc:O0852	Hk2		0	1,744413367	1,15000422	1,354032687	0
ENSMUSG00000025877	Hexokinase-3 (EC 2.7.1.1) (Hexokinase type III) (HK III). [Source:Uniprot/SWISSPROT;Acc:Q3TR	Hk3		0	0,837699659	1,22313912	1,147896779	0,955257357
ENSMUSG00000028961	6-phosphogluconate dehydrogenase, decarboxylating (EC 1.1.1.44). [Source:Uniprot/SWISSPR	Pgd	pentose phosphate Pathway	0	0	0,75903434	0	0
ENSMUSG00000063229	L-lactate dehydrogenase A chain (EC 1.1.1.27) (LDH-A) (LDH muscle subunit) (LDH-M). [Source:	Ldha		0	1,12922393	1,89818424	1,522267929	0
ENSMUSG00000025950	Isocitrate dehydrogenase [NADP] cytoplasmic (EC 1.1.1.42) (Cytosolic NADP-isocitrate dehydro	Idh1	TCA Cycle	0	-1,23202837	-1,09653743	-0,858694783	0
ENSMUSG00000009863	Succinate dehydrogenase [ubiquinone] iron-sulfur subunit, mitochondrial precursor (EC 1.3.5.1	Sdhb	TCA Cycle	0	0	0	-1,057	0
ENSMUSG00000026526	Fumarate hydratase, mitochondrial precursor (EC 4.2.1.2) (Fumarase) (EF-3). [Source:Uniprot/S	Fh1	TCA Cycle	0	0	0	-0,882	0
ENSMUSG00000035493	Transforming growth factor-beta-induced protein ig-h3 precursor (Beta ig-h3). [Source:Uniprot	Tgfbi	Immune Response	0	0	-1,74969764	0	0
ENSMUSG00000026875	TNF receptor-associated factor 1. [Source:Uniprot/SWISSPROT;Acc:P39428]	Traf1		0	0,951411887	0	0	0
ENSMUSG00000023034	Nuclear receptor subfamily 4 group A member 1 (Orphan nuclear receptor HMR) (Nuclear horr	Nr4a1		2,33564923	0	0	0	0
ENSMUSG00000027398	Interleukin-1 beta precursor (IL-1 beta). [Source:Uniprot/SWISSPROT;Acc:P10749]	Il1b		0	0	0	-1,315575005	0
ENSMUSG00000033538	Caspase-4 precursor (EC 3.4.22.64) (CASP-4) (Caspase-11) (CASP-11) (ICH-3 protease) [Contains	Casp4		0	0,807970063	0	0	0
ENSMUSG00000026981	Interleukin-1 receptor antagonist protein precursor (IL-1ra) (IL-1RN) (IRAP) (IL1 inhibitor). [Sou	Il1rn		0,94576245	1,650216475	1,91535962	1,421687394	0
ENSMUSG00000027947	Interleukin-6 receptor alpha chain precursor (IL-6R-alpha) (IL-6R 1) (CD126 antigen). [Source:U	Il6ra		0	-0,92323117	-1,14923195	-0,90027878	0
ENSMUSG00000003882	Interleukin-7 receptor alpha chain precursor (IL-7R-alpha) (CD127 antigen). [Source:Uniprot/SW	Il7r		0	0,804657399	0,93890821	0,749063556	0
ENSMUSG00000024401	Tumor necrosis factor precursor (TNF-alpha) (Tumor necrosis factor ligand superfamily membe	Tnf		1,89763288	2,029073699	1,38263071	0	0
ENSMUSG00000037405	Intercellular adhesion molecule 1 precursor (ICAM-1) (CD54 antigen) (MALA-2) (MyD10). [Sour	Icam1		0	0,850306944	0	0	0
ENSMUSG00000017652	Tumor necrosis factor receptor superfamily member 5 precursor (CD40L receptor) (B-cell surfa	Cd40		0	0	0,94646175	0	0
ENSMUSG00000015396	CD83 antigen [Source:RefSeq_peptide;Acc:NP_033986]	Cd83		1,19204799	0,84842764	0	0	0
ENSMUSG00000022901	T-lymphocyte activation antigen CD86 precursor (Activation B7-2 antigen) (Early T-cell costimu	Cd86		0	0,77526108	0,83923271	0	0
ENSMUSG00000029380	Growth-regulated alpha protein precursor (C-X-C motif chemokine 1) (Platelet-derived growth	Cxcl1	Chemokine	1,54617355	1,098612242	0	0	0
ENSMUSG00000034855	C-X-C motif chemokine 10 precursor (Small-inducible cytokine B10) (Interferon-gamma-induce	Cxcl10		0	0	1,19570684	1,424173848	0
ENSMUSG00000058427	Macrophage inflammatory protein 2 precursor (MIP2) (C-X-C motif chemokine 2). [Source:Unip	Cxcl2		3,25492812	2,101488006	2,33549267	0	0
ENSMUSG00000029379	chemokine (C-X-C motif) ligand 3 [Source:RefSeq_peptide;Acc:NP_976065]	Cxcl3		0	0,938993391	1,18692352	0	0

ENSMUSG00000035385	C-C motif chemokine 2 precursor (Small-inducible cytokine A2) (Monocyte chemotactic protei	Ccl2		1,70111436	0	0	0	0
ENSMUSG00000000982	C-C motif chemokine 3 precursor (Small-inducible cytokine A3) (Macrophage inflammatory pro	Ccl3		2,76824059	2,035651322	1,22715547	0	0
ENSMUSG00000018930	C-C motif chemokine 4 precursor (Small-inducible cytokine A4) (Macrophage inflammatory pro	Ccl4		2,28303004	0	1,05861235	0	0
ENSMUSG00000049103	C-C chemokine receptor type 2 (C-C CKR-2) (CC-CKR-2) (CCR-2) (CCR2) (JE/FIC receptor) (MCP-1	Ccr2		0	-1,6640744	-1,03161793	0	0
ENSMUSG00000043953	C-C chemokine receptor-like 2 (Lipopolysacharide-inducible C-C chemokine receptor) (L-CCR) (	Ccrl2		2,12433766	2,128908878	1,96990509	0	0
ENSMUSG00000020826	Nitric oxide synthase, inducible (EC 1.14.13.39) (NOS type II) (Inducible NO synthase) (Inducibl	Nos2		0	0	2,04860145	2,403772761	0
ENSMUSG00000019987	Arginase-1 (EC 3,5,3,1) (Type I arginase) (Liver-type arginase), [Source:Uniprot/SWISSPROT;Ac	Arg1		0	0	2,037	1,575	1,658
ENSMUSG00000049723	Macrophage metalloelastase precursor (EC 3.4.24.65) (MME) (Matrix metalloproteinase-12) (MMP-12).	Mmp12		0	0	1,602	1,724	1,03
ENSMUSG00000000957	Matrix metalloproteinase-14 precursor (EC 3.4.24.80) (MMP-14) (Membrane-type matrix metalloprotei	Mmp14		0	0,809	0	0	0
ENSMUSG00000029084	ADP-ribosyl cyclase 1 (EC 3.2.2.5) (Cyclic ADP-ribose hydrolase 1) (cADPr hydrolase 1) (NIM-R5 antigen) (	Cd38	Adhesion molecules	0	0	0,832	0,74	0
ENSMUSG00000000555	Integrin alpha-5 precursor (Fibronectin receptor subunit alpha) (Integrin alpha-F) (VLA-5) (CD49e antiger	Itga5		0	0,924094724	0,720036034	0,825394513	0
ENSMUSG00000037405	Intercellular adhesion molecule 1 precursor (ICAM-1) (CD54 antigen) (MALA-2) (MyD10). [Source:Unipro	Icam1		0	0,931	0	0	0
ENSMUSG00000021670	3-hydroxy-3-methylglutaryl-coenzyme A reductase (EC 1.1.1.34) (HMG-CoA reductase). [Sourc	Hmgcr	Chholesterol pathway	0	0,766	0	0	0
ENSMUSG00000022351	Squalene monooxygenase (EC 1.14.99.7) (Squalene epoxidase) (SE). [Source:Uniprot/SWISSPR	Sqle		0	0,810897603	0	0	0
ENSMUSG00000026170	Cytochrome P450 27, mitochondrial precursor (EC 1.14.13.15) (Cytochrome P-450C27/25) (Ste	Cyp27a1		0	-0,7250079	-1,44239693	-1,272885117	0
ENSMUSG00000015243	ATP-binding cassette sub-family A member 1 (ATP-binding cassette transporter 1) (ATP-bindin	Abca1		0	0	-0,93577279	0	0
ENSMUSG00000025203	Acyl-CoA desaturase 2 (EC 1.14.19.1) (Stearoyl-CoA desaturase 2) (Fatty acid desaturase 2) (De	Scd2		0	0	0	1,521323372	1,515090128
ENSMUSG00000002944	Platelet glycoprotein 4 (Platelet glycoprotein IV) (GPIV) (GPIIIB) (CD36 antigen) (PAS IV) (PAS-4	Cd36		0	0	1,33190094	0	0
ENSMUSG00000007655	Caveolin-1. [Source:Uniprot/SWISSPROT;Acc:P49817]	Cav1		0	0	0	0,745881736	0
ENSMUSG00000022305	Low-density lipoprotein receptor-related protein 12 precursor. [Source:Uniprot/SWISSPROT;A	Lrp12		0	1,043802329	0	0	0
ENSMUSG00000076435	Acyl-CoA synthetase family member 2, mitochondrial precursor (EC 6.2.1.-). [Source:Uniprot/S	Acsf2		0	0	-0,72716511	0	0
ENSMUSG00000018796	Long-chain-fatty-acid--CoA ligase 1 (EC 6.2.1.3) (Long-chain acyl-CoA synthetase 1) (LACS 1). [S	Acsl1		0	1,305541141	1,1225562	1,286080999	0
ENSMUSG00000062515	Fatty acid-binding protein, adipocyte (AFABP) (Adipocyte lipid-binding protein) (ALBP) (A-FABI	Fabp4		0	1,478791089	1,68120804	0	0
ENSMUSG00000015568	Lipoprotein lipase precursor (EC 3.1.1.34) (LPL). [Source:Uniprot/SWISSPROT;Acc:P11152]	Lpl		0	0	0	0	0,794
ENSMUSG00000029314	1-acyl-sn-glycerol-3-phosphate acyltransferase theta (EC 2.3.1.51) (Lysophosphatidic acid acylt	Agpat9	Triglyceride pathway	0	0,724642752	0,71502502	0	0
ENSMUSG00000028517	Lipid phosphate phosphohydrolase 3 (EC 3.1.3.4) (Phosphatidic acid phosphatase 2b) (Phospha	Ppap2b		0	1,052284718	1,26225361	1,112020775	0
ENSMUSG00000030747	Diacylglycerol O-acyltransferase 2 (EC 2.3.1.20) (Diglyceride acyltransferase 2). [Source:Unipro	Dgat2		0	0,845	0	0	0
ENSMUSG00000031467	1-acyl-sn-glycerol-3-phosphate acyltransferase epsilon (EC 2.3.1.51) (1-AGP acyltransferase 5)	Agpat5		0	0,802476155	0	0	0
ENSMUSG00000056220	Cytosolic phospholipase A2 (cPLA2) (Phospholipase A2 group IVA) [Includes: Phospholipase A2	Pla2g4a	Prostaglandine synthesis	0	0	0,7902618	0,717374986	0
ENSMUSG00000028378	NADP-dependent leukotriene B4 12-hydroxydehydrogenase (EC 1.3.1.74) (15-oxoprostaglandi	Ltb4dh		0	0	1,21089194	0	0
ENSMUSG00000029919	Glutathione-requiring prostaglandin D synthase (EC 5.3.99.2) (Glutathione-dependent PGD syr	Ptgds2		0	0	0	-0,806259096	0
ENSMUSG00000039942	Prostaglandin E2 receptor EP4 subtype (Prostanoid EP4 receptor) (PGE receptor, EP4 subtype).	Ptger4		0,82848723	0	0	0	0
ENSMUSG00000050737	Prostaglandin E synthase (EC 5.3.99.3) (mPGES-1). [Source:Uniprot/SWISSPROT;Acc:Q9JM51]	Ptges		0	1,318719911	1,78705281	1,599546431	0
ENSMUSG00000071072	Prostaglandin E synthase 3 (EC 5.3.99.3) (Cytosolic prostaglandin E2 synthase) (cPGES) (Telome	Ptges3		0	0	0,93676353	0	0
ENSMUSG00000047250	Prostaglandin G/H synthase 1 precursor (EC 1.14.99.1) (Cyclooxygenase- 1) (COX-1) (Prostaglai	Ptgs1 (cox1)		0	0	-1,31384103	-1,279805241	0
ENSMUSG00000032487	Prostaglandin G/H synthase 2 precursor (EC 1.14.99.1) (Cyclooxygenase- 2) (COX-2) (Prostaglai	Ptgs2 (cox2)		1,95483853	2,240586175	1,78799857	2,459334673	0
ENSMUSG00000016427	NADH dehydrogenase [ubiquinone] 1 alpha subcomplex subunit 1 (NADH- ubiquinone oxidore	Ndufa1	Complex I structure	0	0	0,977	-0,863	0
ENSMUSG00000014294	NADH dehydrogenase [ubiquinone] 1 alpha subcomplex subunit 2 (NADH- ubiquinone oxidore	Ndufa2		0	0	0,917	-0,892	0
ENSMUSG00000023089	NADH dehydrogenase [ubiquinone] 1 alpha subcomplex subunit 5 (NADH- ubiquinone oxidore	Ndufa5		0	0	0	-0,954	0
ENSMUSG00000026895	NADH dehydrogenase [ubiquinone] 1 alpha subcomplex subunit 8 (NADH- ubiquinone oxidore	Ndufa8		0	0	0	-0,745	0
ENSMUSG00000040048	NADH dehydrogenase [ubiquinone] 1 beta subcomplex subunit 10 (NADH- ubiquinone oxidore	Ndufb10		0	0	0	-0,816	0
ENSMUSG00000031059	NADH dehydrogenase [ubiquinone] 1 beta subcomplex subunit 11, mitochondrial precursor (N	Ndufb11		0	0	0	-0,928	0

ENSMUSG00000002416	NADH dehydrogenase [ubiquinone] 1 beta subcomplex subunit 2, mitochondrial precursor (NA	Ndufb2		0	0	0	-0,864	0
ENSMUSG00000025204	NADH dehydrogenase [ubiquinone] 1 beta subcomplex subunit 8, mitochondrial precursor (NA	Ndufb8		0	0	0	-0,887	0
ENSMUSG00000030647	NADH dehydrogenase [ubiquinone] 1 subunit C2 (NADH-ubiquinone oxidoreductase subunit B	Ndufc2		0	0	0	-1,114	0
ENSMUSG00000009863	Succinate dehydrogenase [ubiquinone] iron-sulfur subunit, mitochondrial precursor (EC 1.3.5.1	Sdhb	Complex II activity + Sdhb (T	0	0	0	-1,057	0
ENSMUSG00000059534	Cytochrome b-c1 complex subunit 9 (Ubiquinol-cytochrome c reductase complex 7.2 kDa prote	Uqcr10	Complex III-May function as	0	0	0,834	-0,897	0
ENSMUSG00000038462	Cytochrome b-c1 complex subunit Rieske, mitochondrial precursor (EC 1.10.2.2) (Ubiquinol-cyt	Uqcrfs1	Complex III-Nuclear subunit-	0	0	0	-0,965	0
ENSMUSG00000063882	Cytochrome b-c1 complex subunit 6, mitochondrial precursor (Ubiquinol- cytochrome c reduct	Uqcrh	Complex III-UQCRH (Subunit	0	0	0,772	0	0
ENSMUSG00000046516	Cytochrome c oxidase copper chaperone. [Source:Uniprot/SWISSPROT;Acc:P56394]	Cox17	Complex IV	0	0	0,893	0	0
ENSMUSG00000000088	Cytochrome c oxidase subunit 5A, mitochondrial precursor (Cytochrome c oxidase polypeptide	Cox5a		0	0	0,793	-1,119	0
ENSMUSG00000041697	Cytochrome c oxidase polypeptide VIa-liver, mitochondrial precursor. [Source:Uniprot/SWISSP	Cox6a1		0	0	0	-1,24	0
ENSMUSG00000036751	Cytochrome c oxidase subunit VIb isoform 1 (COX VIb-1). [Source:Uniprot/SWISSPROT;Acc:P56	Cox6b1		0	0	0,818	-1,098	0
ENSMUSG00000031818	Cytochrome c oxidase subunit 4 isoform 1, mitochondrial precursor (Cytochrome c oxidase sub	Cox4i1		0	0	0,834	-1,27	0
ENSMUSG00000024248	Cytochrome c oxidase subunit VIIa-related protein, mitochondrial precursor (Silica-induced gen	Cox7a2l		0	0	0	-1,565	0
ENSMUSG00000016252	ATP synthase subunit epsilon, mitochondrial. [Source:Uniprot/SWISSPROT;Acc:P56382]	Atp5e	Peripheral moiety: F1 /Nucl	0	0	0	-1,298	0
ENSMUSG00000018770	ATP synthase lipid-binding protein, mitochondrial precursor (ATP synthase proteolipid P3) (AT	Atp5g3	Integral membrane compone	0	0	1,142	-1,261	0
