# Supplementary material for: Transcriptomic Signature of Leishmania Infected Mice Macrophages: A Metabolic Point of View
Source: PLoS Negl Trop Dis. 2012 Aug 21;6(8):e1763. doi: 10.1371/journal.pntd.0001763 (PMC3424254; doi:10.1371/journal.pntd.0001763)
Supplement: Table S3 — Selected genes up- or down-regulated more than two-fold in Leishmania infected BMdM as measured by qRT-PCR. Genes selected in Table S2 were tested by qRT-PCR. Changes in mRNA levels are calculated using the 2−ΔΔCT method. The numbers presented for each time point are the average of the three biological replicates. (PDF) [file pntd.0001763.s003.pdf]

Table S3 : selected genes up- or down-regulated more than two-fold in Leishmania infected BMdM as confirmed by qPCR

Ensembl.Gene.ID	Description	MGI Symbol	Pathways	2-DDCt (1h)	2-DDCt (3h)	2-DDCt (6h)	2-DDCt (12h)	2-DDCt (24h)
ENSMUSG00000028645	Solute carrier family 2, facilitated glucose transpor	Slc2a1	Glycolysis pathway	1,045465171	6,2372064	5,4248597	2,33128034	1,94642713
ENSMUSG00000036427	Glucose-6-phosphate isomerase (EC 5.3.1.9) (GPI) (	Gpi1		0,952787682	1,21986655	1,7652153	1,50440621	1,78402603
ENSMUSG00000020277	6-phosphofructokinase, liver type (EC 2.7.1.11) (Ph	Pfkl		0,864612391	2,03009185	2,7587704	0,97192372	1,04108835
ENSMUSG00000021196	6-phosphofructokinase type C (EC 2.7.1.11) (Phosp	Pfkp		1,146454223	2,81047049	5,0384865	1,6289662	1,67585133
ENSMUSG00000030695	aldolase 1, A isoform, retrogene 1 [Source:MGI;Ac	Aldoa		0,895661449	1,27286846	2,4430026	1,08567829	2,14165956
ENSMUSG00000017390	Fructose-bisphosphate aldolase C (EC 4.1.2.13) (Br	Aldoc		2,175042739	2,50384166	0,8238444	3,87504125	2,09395315
ENSMUSG00000023456	Triosephosphate isomerase (EC 5.3.1.1) (TIM) (Trio	Tpi1		0,836861046	1,62932191	3,3771334	1,38504466	2,05509488
ENSMUSG00000004267	Gamma-enolase (EC 4.2.1.11) (2-phospho-D-glycer	Eno2		1,26746896	3,324122	3,1585602	1,33751493	1,94188016
ENSMUSG00000032294	Pyruvate kinase isozymes M1/M2 (EC 2.7.1.40) (Py	Pkm2		0,909676454	1,2957224	2,3225727	1,15663741	1,79762551
ENSMUSG00000057666	Glyceraldehyde-3-phosphate dehydrogenase (EC 1	Gapdh		0,964361136	1,24320424	2,3675563	1,26701769	1,97560451
ENSMUSG00000026773	6-phosphofructo-2-kinase/fructose-2,6-biphospha	Pfkfb3		1,279124478	2,42577095	2,0562692	1,68223101	1,28495544
ENSMUSG00000006494	Pyruvate dehydrogenase [lipoamide]] kinase isozy	Pdk1		0,683282342	1,56646949	2,6069666	1,2655095	1,71797515
ENSMUSG00000022707	1,4-alpha-glucan-branching enzyme (EC 2.4.1.18) ((	Gbe1	strach degradation	0,861982459	1,8640945	8,6470044	2,15351743	2,05673487
ENSMUSG00000025791	Phosphoglucomutase-1 (EC 5.4.2.2) (Glucose phos	Pgm2		0,851560931	2,64930388	4,796967	1,88165765	1,83737572
ENSMUSG00000037012	Hexokinase-1 (EC 2.7.1.1) (Hexokinase type I) (HK I	Hk1		1,633415323	0,17199219	0,6965622	1,26921297	0,29806558
ENSMUSG00000000628	Hexokinase-2 (EC 2.7.1.1) (Hexokinase type II) (HK	Hk2		1,490569609	2,40349144	2,6472915	1,08483954	1,51731872
ENSMUSG00000028961	6-phosphogluconate dehydrogenase, decarboxylat	Pgd	pentose phosphate Pathway	0,910371364	0,97108535	1,7111747	0,86234974	0,86167008
ENSMUSG00000031299	Apyruvate dehydrogenase E1 alpha 1	Pdha1		0,944281395	0,95325229	0,9112573	0,69385206	1,23596895
ENSMUSG00000063229	L-lactate dehydrogenase A chain (EC 1.1.1.27) (LDH	Ldha		0,87189446	2,11647728	3,9838558	1,223898	1,96372671
ENSMUSG00000024892	pyruvate carboxylase	Pcx		0,786519806	0,99518272	1,938312	0,95148149	0,99347576
ENSMUSG00000046934	citrate synthase like	Csl		0,921782798	0,66293837	1,0483124	0,65477675	0,86390775
ENSMUSG00000025950	Isocitrate dehydrogenase [NADP] cytoplasmic (EC 1	Idh1	TCA Cycle	0,907770808	0,5196048	0,5301531	0,6496941	0,76087692
ENSMUSG00000021577	succinate dehydrogenase complex, subunit A, flav	Sdha	TCA Cycle	0,8638566	0,83795325	0,7296334	0,57782452	0,9180847
ENSMUSG00000009863	Succinate dehydrogenase [ubiquinone] iron-sulfur	Sdhb	TCA Cycle	0,863756809	0,83719175	0,7720879	0,56190767	0,93364597
ENSMUSG00000026526	Fumarate hydratase, mitochondrial precursor (EC 4	Fh1	TCA Cycle	0,746804918	0,65581009	0,4407001	0,75230304	0,9015462
ENSMUSG00000035493	Transforming growth factor-beta-induced protein i	Tgfbi		1,021392002	0,54483137	0,7244949	1,10604813	0,73927942
ENSMUSG00000023034	Nuclear receptor subfamily 4 group A member 1 ((	Nr4a1		8,694782868	1,98167448	1,5079018	1,68482743	0,38218905
ENSMUSG00000027398	Interleukin-1 beta precursor (IL-1 beta). [Source:Ur	Il1b		2,002321078	0,97034409	0,8021001	0,7661148	0,17035289

ENSMUSG00000026981	Interleukin-1 receptor antagonist protein precurso	Il1rn	Immune Response	2,256296503	3,60939408	6,6618175	4,10228436	2,48251655
ENSMUSG00000027947	Interleukin-6 receptor alpha chain precursor (IL-6R	Il6ra		0,84007983	0,43589876	0,4682554	0,73154364	0,69668416
ENSMUSG00000003882	Interleukin-7 receptor alpha chain precursor (IL-7R	Il7r		0,897995684	1,65192898	1,6961657	1,03205749	1,23806967
ENSMUSG00000024401	Tumor necrosis factor precursor (TNF-alpha) (Tum	Tnf		3,492362543	3,9715211	3,1793735	5,45256791	1,27157363
ENSMUSG00000037405	Intercellular adhesion molecule 1 precursor (ICAM	Icam1		1,173991259	1,74824414	1,4671155	1,24674161	0,9493476
ENSMUSG00000017652	Tumor necrosis factor receptor superfamily memb	Cd40		1,538844104	2,94777212	5,7281082	3,18370501	0,33294386
ENSMUSG00000015396	CD83 antigen [Source:RefSeq_peptide;Acc:NP_033	Cd83		1,813877571	3,81571477	0,9129211	1,07480629	0,49828308
ENSMUSG00000022901	T-lymphocyte activation antigen CD86 precursor (A	Cd86		1,280158179	1,67136288	1,2409575	1,43351964	0,35752371
ENSMUSG00000029380	Growth-regulated alpha protein precursor (C-X-C n	Cxcl1	Chemokine	6,370862718	4,97805901	2,4932578	4,56614498	0,63834614
ENSMUSG00000034855	C-X-C motif chemokine 10 precursor (Small-induci	Cxcl10		1,236915261	1,01702607	3,1058665	3,98197501	1,23422892
ENSMUSG00000058427	Macrophage inflammatory protein 2 precursor (MI	Cxcl2		6,766385701	5,36028608	2,3663231	3,94840506	0,5054134
ENSMUSG00000029379	chemokine (C-X-C motif) ligand 3 [Source:RefSeq_	Cxcl3		2,733631448	2,27156775	3,870072	2,33694428	0,89343468
ENSMUSG00000035385	C-C motif chemokine 2 precursor (Small-inducible	Ccl2		5,497783137	1,75884904	0,9529489	2,38298108	0,72422255
ENSMUSG00000000982	C-C motif chemokine 3 precursor (Small-inducible	Ccl3		4,173247305	2,97367779	1,5566761	2,48272018	1,25607926
ENSMUSG00000018930	C-C motif chemokine 4 precursor (Small-inducible	Ccl4		4,327464796	4,1754017	2,3753752	3,09121357	0,91452161
ENSMUSG00000049103	C-C chemokine receptor type 2 (C-C CKR-2) (CC-CKF	Ccr2		0,94778304	0,40860016	0,5841687	0,67883209	0,65185553
ENSMUSG00000043953	C-C chemokine receptor-like 2 (Lipopolysacharide-	Ccrl2		2,132965183	4,31354299	2,6930427	1,50905808	0,77614198
ENSMUSG00000020826	Nitric oxide synthase, inducible (EC 1.14.13.39) (NC	Nos2		1,58014697	6,058350306	47,5176665	7,213036212	6,18884776
ENSMUSG00000019987	Arginase-1 (EC 3,5,3,1) (Type I arginase) (Liver-type	Arg1		0,755111756	1,310101268	5,9167793	1,535726883	0,863264261
ENSMUSG00000021670	3-hydroxy-3-methylglutaryl-coenzyme A reductase	Hmgcr	Chholesterol pathway	1,357089074	1,55569222	1,4248981	0,83228719	0,8671404
ENSMUSG00000022351	Squalene monooxygenase (EC 1.14.99.7) (Squalene	Sqle		1,329481544	2,5228714	3,1179413	1,45305737	1,4464193
ENSMUSG00000026170	Cytochrome P450 27, mitochondrial precursor (EC	Cyp27a1		0,846888141	0,66029304	0,1711296	0,44647279	0,46220881
ENSMUSG00000015243	ATP-binding cassette sub-family A member 1 (ATP	Abca1		0,851528713	0,87721051	0,6734738	0,83446879	0,75744647
ENSMUSG00000025203	Acyl-CoA desaturase 2 (EC 1.14.19.1) (Stearoyl-CoA	Scd2		0,939264464	1,00803565	1,7796816	1,86733292	2,87151746
ENSMUSG00000002944	Platelet glycoprotein 4 (Platelet glycoprotein IV) (G	Cd36		0,84873167	1,43561609	2,2866418	1,96686781	2,4853933
ENSMUSG00000007655	Caveolin-1. [Source:Uniprot/SWISSPROT;Acc:P498	Cav1		0,967616371	1,01872289	2,1620558	1,33890011	0,67451425
ENSMUSG00000022305	Low-density lipoprotein receptor-related protein 1	Lrp12		0,934865301	1,70524811	1,4245047	0,82500037	1,09362073
ENSMUSG00000018796	Long-chain-fatty-acid--CoA ligase 1 (EC 6.2.1.3) (Lo	Acsl1		1,12963661	2,15349753	3,6231773	1,43883717	1,39693591
ENSMUSG00000062515	Fatty acid-binding protein, adipocyte (AFABP) (Adi	Fabp4		1,21260142	2,20402156	1,9952904	1,21761525	1,21945301
ENSMUSG00000015568	Lipoprotein lipase precursor (EC 3.1.1.34) (LPL). [Sc	Lpl		0,6334578	0,87081309	0,9258062	1,68456372	2,94850948
ENSMUSG00000028517	Lipid phosphate phosphohydrolase 3 (EC 3.1.3.4) (l	Ppap2b		1,161150519	4,35687382	7,4215173	2,22154367	2,34115156
ENSMUSG00000030747	Diacylglycerol O-acyltransferase 2 (EC 2.3.1.20) (Di	Dgat2		0,764091376	2,69966426	1,2700354	0,82693739	0,80381245

ENSMUSG00000031467 1-acyl-sn-glycerol-3-phosphate acyltransferase eps	Agpat5		0,902617768	2,19808676	1,8327929	0,96308386	1,02835793
ENSMUSG00000056220 Cytosolic phospholipase A2 (cPLA2) (Phospholipas	Pla2g4a	Prostaglandine synthesis	1,080155299	2,33603194	4,1006438	1,51134532	1,29621572
ENSMUSG00000028378 NADP-dependent leukotriene B4 12-hydroxydehyc	Ltb4dh		0,917572038	1,45797232	3,7112559	1,7832729	1,56575303
ENSMUSG00000029919 Glutathione-requiring prostaglandin D synthase (E	Ptgds2		0,864727265	1,53899833	0,640017	0,4710319	0,81495982
ENSMUSG00000039942 Prostaglandin E2 receptor EP4 subtype (Prostanoic	Ptger4		2,277980531	1,18797406	0,7858262	1,07334956	1,02092072
ENSMUSG00000050737 Prostaglandin E synthase (EC 5.3.99.3) (mPGES-1).	Ptges		1,402295521	3,98403411	17,936259	3,63539804	2,75078967
ENSMUSG00000071072 Prostaglandin E synthase 3 (EC 5.3.99.3) (Cytosolic	Ptges3		1,045249304	1,35860085	1,3767059	0,71889435	0,85888537
ENSMUSG00000047250 Prostaglandin G/H synthase 1 precursor (EC 1.14.9	Ptgs1 (cox1)		0,976846478	0,36452466	0,1209849	0,30995056	0,45007921
ENSMUSG00000032487 Prostaglandin G/H synthase 2 precursor (EC 1.14.9	Ptgs2 (cox2)		8,396377802	5,3499114	10,167681	4,93440113	1,89403873
ENSMUSG00000014294 NADH dehydrogenase [ubiquinone] 1 alpha subcor	Ndufa2		1,018525488	0,94964816	1,0588308	0,75887456	1,01559545
ENSMUSG00000030647 NADH dehydrogenase [ubiquinone] 1 subunit C2 (I	Ndufc2		1,245203972	0,85666814	1,0482071	0,64795851	0,89338308
ENSMUSG00000021764 NADH dehydrogenase [ubiquinone] iron-sulfur pro	Ndufs4	Complex I activit	0,928156775	0,94779153	1,0479153	0,68223153	0,96510256
ENSMUSG00000021577 succinate dehydrogenase complex, subunit A, flav	Sdha	Complex II activi	0,8638566	0,83795325	0,7296334	0,57782452	0,9180847
ENSMUSG00000009863 Succinate dehydrogenase [ubiquinone] iron-sulfur	Sdhb	Complex II activi	0,863756809	0,83719175	0,7720879	0,56190767	0,93364597
ENSMUSG00000038462 Cytochrome b-c1 complex subunit Rieske, mitocho	Uqcrfs1	Complex III-Nucl	1,122525914	1,27464898	0,5723146	0,6526667	1,01308075
ENSMUSG00000063882 Cytochrome b-c1 complex subunit 6, mitochondria	Uqcrh	Complex III-UQC	0,911764883	0,98165864	0,9828867	0,79170599	1,02519321
ENSMUSG00000046516 Cytochrome c oxidase copper chaperone. [Source:I	Cox17	Complex IV	1,265625363	1,29685603	1,1890344	0,68976173	1,05083476
ENSMUSG00000000088 Cytochrome c oxidase subunit 5A, mitochondrial p	Cox5a		1,01269314	1,08244721	0,8722833	0,62876377	0,87795922
ENSMUSG00000036751 Cytochrome c oxidase subunit VIb isoform 1 (COX V	Cox6b1		0,926850356	0,98443419	0,8445419	0,77913083	0,98206698
ENSMUSG00000031818 Cytochrome c oxidase subunit 4 isoform 1, mitoch	Cox4i1		0,895161824	0,99824672	0,854801	0,65967317	0,96013832
ENSMUSG00000024248 Cytochrome c oxidase subunit VIIa-related protein	Cox7a2l		1,014769221	0,92969109	0,7199494	0,88301764	0,97024433
ENSMUSG00000016252 ATP synthase subunit epsilon, mitochondrial. [Sou	Atp5e	Peripheral moiet	0,963568242	0,93618464	0,8721614	0,65444098	0,93261756
ENSMUSG00000018770 ATP synthase lipid-binding protein, mitochondrial	Atp5g3	Integral membra	0,995108276	1,12222607	1,0492841	0,67829354	0,893891
